# Multi‐Axis Stretchable Zippers for Personalized Wound Healing

**DOI:** 10.1002/advs.75744

**Published:** 2026-06-11

**Authors:** Siyuan Cai, Guang Yao, Zijian Chen, Shiqi Zhou, Peisi Li, Liheng Lin, Huake Yang, Ziyi Zhou, Linbo Jin, Xingyi Gan, Chenzheng Zhou, Zhen Cai, Taisong Pan, Min Gao, Dongli Fan, Yuan Lin, Yiming Zhang

**Affiliations:** ^1^ Department of Plastic and Cosmetic Surgery Xinqiao Hospital Army Medical University Chongqing China; ^2^ School of Materials and Energy University of Electronic Science and Technology of China Chengdu Sichuan China; ^3^ State Key Laboratory of Electronic Thin Films and Integrated Devices University of Electronic Science and Technology of China Chengdu Sichuan China; ^4^ Department of Plastic Surgery Sichuan Provincial People's Hospital University of Electronic Science and Technology of China Chengdu Sichuan China; ^5^ Shenzhen Institute for Advanced Study University of Electronic Science and Technology of China Shenzhen China; ^6^ Medico‐Engineering Cooperation on Applied Medicine Research Center University of Electronic Science and Technology of China Chengdu Sichuan China; ^7^ Department of Anesthesiology The First People's Hospital of Longquanyi District Chengdu Sichuan China

## Abstract

Effective and controlled mechanical wound closure is essential for preventing infection, promoting re‐epithelialization, and minimizing scarring. However, existing wound closure technologies are limited to uniaxial closures and lack programmability, which hinders their adaptability to wounds with complex morphologies and restricts personalized treatment needs. Here, we propose a multiaxial stretchable wound zipper engineered from electrothermally driven mechanical metamaterials. The device features a hierarchical lattice of shape memory alloys, enabling six axes of stretching and programmable contraction via a smartphone. It delivers adjustable contraction force ranging from 0 to 0.494 MPa, adaptable to diverse wound geometries, with a rapid response time of approximately 1.73 s. In the rat model, the device achieved near‐instantaneous closure of linear wounds and improved the circular wound‐healing rate by 35.91% compared with the control group. Mechanistically, the programmable mechanical contraction promoted vascular regeneration, re‐epithelialization, and collagen matrix remodeling, ultimately accelerating personalized wound healing. The device achieved rapid, robust, and programmable multiaxial contraction, demonstrating substantial potential for personalized wound management and clinical translation.

## Graphical abstract

Multi‐axis stretchable zippers can adapt to complex wound morphologies and enable personalized wound treatment through programmable contraction. This morphological matching strategy helps accelerate wound closure, improve blood perfusion, promote epithelial regeneration, and enhance collagen remodeling, pioneering new directions for personalized and precise wound treatment.

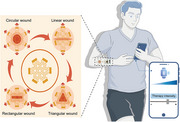

## 1 Introduction

In addition to thermoregulation and environmental sensing, the skin serves as the body's primary barrier against external aggression [1, 2]. Due to continuous exposure to the external environment, the skin is susceptible to injury and wound formation, imposing a heavy burden on the global public health system [3, 4, 5]. Following wound formation, prompt and effective mechanical closure is crucial for infection control, accelerated reepithelialization, and scar reduction [6, 7]. Emphasis should be placed on the fact that the skin is a highly specialized mechanosensory organ, exceptionally sensitive to mechanical forces [8]. A large body of evidence indicates that moderate mechanical stimulation can not only accelerate wound healing but also promote cell proliferation, collagen deposition, and angiogenesis by modulating the matrix‐microenvironment, resulting in higher quality tissue regeneration [9, 10, 11, 12]. Therefore, mechanical force is regarded as a core regulatory factor in the wound repair process, which is a key link to promote skin regeneration and tissue remodeling [8].

Conventional mechanical closure methods, including surgical sutures, dermal staples, and wound zippers, are unidirectional and primarily suited for linear wounds without significant skin defects [13, 14, 15]. Their adaptability to geometrically complex wounds with skin defects is limited [16]. Furthermore, these techniques heavily rely on manual handling by professionals, which is not conducive to rapid treatment in emergencies [17]. In recent years, stimulus‐responsive contractile dressings—which actively close wounds by sensing external stimuli to trigger volume changes or phase transitions—have emerged as a promising alternative [18]. Such systems typically rely on deformation mechanisms involving hydrogels [19], polymeric shrink films [20], shape‐memory polymers [21], or liquid crystal elastomers [22]; most require activation by temperature or humidity, making them susceptible to environmental factors, and generally exhibit low contractile force and efficiency [17, 23]. Additionally, their contraction process is unpredictable, making it difficult to accommodate differences in patient tolerance (e.g., diabetic patients may have reduced pain sensitivity due to peripheral neuropathy, while others may be more sensitive to the contractile force [24]). Such dressings lack designs tailored to the complex morphologies of wounds, making them prone to mechanical mismatch and localized stress concentration, thereby increasing the risk of tissue damage [25, 26]. Their limitations are evident: weak and inefficient contraction, non‐adjustable and difficult to quantify, and poor adaptability to complex wound geometries. Advances in flexible bioelectronic systems (FBEs) offer new approaches to active wound contraction [27, 28, 29]. FBEs combine excellent skin conformability with the ability to integrate micro‐control systems, enabling precise regulation of therapeutic parameters [30, 31]. For example, the integration of flexible electrodes with microelectronic modules can provide continuous electrical stimulation and support real‐time parameter adjustment [32, 33]; Kang et al. achieved programmable, spatially controllable mechanical output by integrating mechanical components [34]. These studies demonstrate the feasibility of integrating functional materials into FBEs. To our knowledge, existing research on mechanical actuation of FBEs primarily focuses on sensing applications such as haptic feedback and has not yet been applied to wound closure. Overall, FBEs demonstrate significant potential for achieving highly efficient, programmable mechanical contraction and adapting to complex wound morphologies, and are expected to advance personalized and precise wound treatment.

Here, we propose and validate a multi‐axis stretchable wound zipper (MSWZ) based on electro‐thermally driven mechanical metamaterials. The device offers flexibility stretched along six axes, spaced 60 degrees apart, enabling precise adaptation to complex wound morphologies. Upon pre‐stretching, the device stably stores mechanical potential energy and rapidly contracts via modulus‐matched stretchable circuits, exhibiting a minimum response time of ∼1.73 s. The MSWZ delivers programmable mechanical contraction forces tailored to different wound morphologies: 0–0.494 MPa for linear wounds, 0–0.274 MPa for triangular wounds, 0–0.298 MPa for rectangular wounds, and 0–0.243 MPa for circular wounds. The corresponding achieved wound closure rates were 100%, 84.85%, 81.99%, and 87.40% for linear, triangular, rectangular, and circular wounds, respectively. The in vivo MSWZ verification was performed using a Sprague–Dawley (SD) rat model. In the linear wound model, the MSWZ achieved immediate and complete closure, with a wound‐healing rate of nearly 90% on day 1. In the circular skin defect model, MSWZ showed a superior wound‐healing rate (> 95.1%) within 8 days, representing 35.9% improvement compared to the control group. Mechanistically, the mechanical contraction of MSWZ upregulated vascular regeneration‐related transcription factors and accelerated local perfusion restoration. Concurrently, it promoted proliferation and migration of keratinocytes, thereby expediting re‐epithelialization. In addition, by reducing the skin tension at the wound margin, the MSWZ inhibited the over‐activation of myofibroblasts, facilitated orderly collagen matrix remodeling, and decreased the risk of scar formation. This strategy achieves rapid, robust, and programmable multiaxial mechanical wound closure, establishing a solid basis for personalized wound management and subsequent clinical translation.

## 2 Results and Discussion

### 2.1 Design and Treatment Principle of MSWZ

**FIGURE 1:**
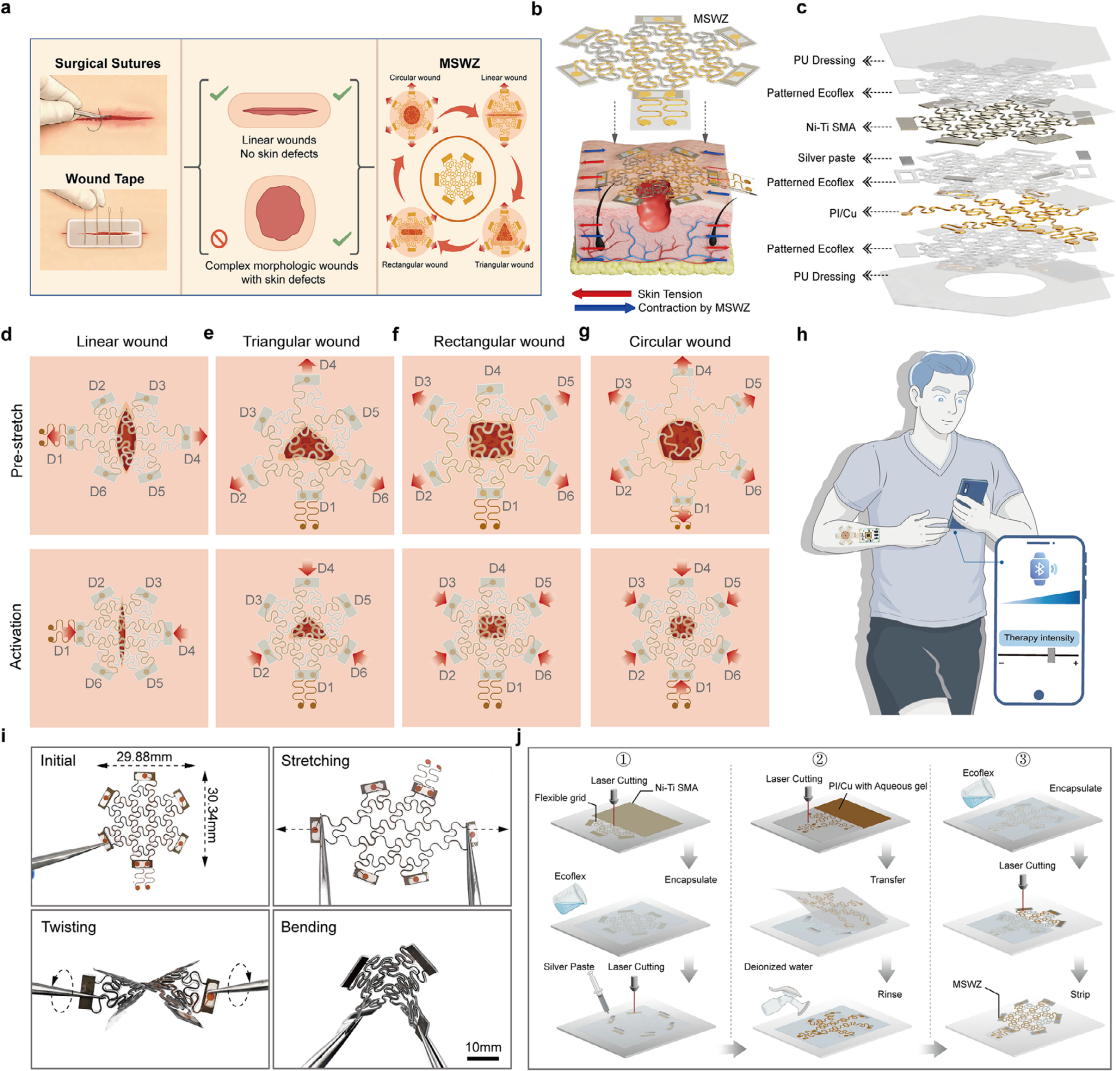
Design and therapeutic principles of MSWZ. (a) Multi‐axial stretchability of MSWZ matches complex wound morphologies. (b) MSWZ can be stably applied to the skin and effectively closes the wound. (c) Exploded diagram of the structure of the MSWZ. The MSWZ is pre‐stretched and then activated to contract along six axes for different wound conditions, including: (d) linear wound, (e) triangular wound, (f) rectangular wound, and (g) circular wound. (h) The patient modulates the MSWZ to treat the wound via a mobile application. (i) The MSWZ undergoes stretching, twisting & bending and still returns to its initial state. (j) Detailed manufacturing process of MSWZ.

Traditional surgical suture techniques or wound zippers are only applicable to linear wounds without skin defects and are difficult to cope with complex wound morphologies with skin defects. Moreover, these traditional methods are cumbersome to operate, with limited and difficult to quantify precision of modulation. MSWZ can be freely stretched along multiple axes to adapt to different morphologies and sizes of wounds. When activated, the MSWZ automatically closes the wound edge like a zipper, and its mechanical contraction is programmable and easy to operate (Figure 1a). The MSWZ possesses excellent flexibility and ductility, allowing for a stable and seamless fit to the skin surface and closure of the wound by applying mechanical contraction (Figure 1b).

As shown in Figure 1c, the MSWZ adopts a mechanical metamaterial based on nickel–titanium shape memory alloy (Ni–Ti SMA) as the core shrinkage unit in its structural design. The metamaterial consists of a hierarchical lattice structure, which has a “J‐shaped” nonlinear mechanical response similar to that of human skin, and can provide biomimetic mechanical protection during the contraction process [35, 36]. When triggered by an electro‐thermal drive, the Ni–Ti SMA undergoes a reversible phase transition from martensite to austenite, resulting in a fast and stable electro‐mechanical response [37]. The device employs a serpentine polyimide/copper (PI/Cu) conductive layer to ensure reliability under high strain conditions. This structure maintains a stable electrical connection under large deformations while avoiding strain limitation on the lattice. The outer encapsulation material is a skeletonized Ecoflex elastomer, which further enhances the system's superelastic properties and breathability while maintaining high stretchability. The detailed principle of MSWZ's electrothermal mechanical shrinkage is shown in Figure S1. The MSWZ is encapsulated between two layers of polyurethane (PU) dressing before use. A circular notch with a radius of approximately 10 mm in the center of the underlying PU dressing allows the MSWZ to adhere only to the wound margins and not to the center of the wound, thus avoiding mechanical contraction forces acting directly on the central wound tissue. This “loop adhesion” design is superior to traditional homogeneous dressing structures and allows the MSWZ to be activated to draw flat against the wound edge like a bag of drawstrings for a smoother, more uniform closure process [9]. The MSWZ can be flexibly stretched in six directions (D1‐D6) spaced at 60° intervals to accommodate different wound morphologies and sizes. Pre‐stretching along the D1 and D4 directions matches linear wounds (Figure 1d); along the D2, D4, and D6 directions matches triangular wounds (Figure 1e); along the D2, D3, D5, and D6 directions matches rectangular wounds (Figure 1f); and stretching along the D1‐D6 directions matches circular wounds (Figure 1g). The MSWZ achieves rapid and precise mechanical closure when activated by the stretchable circuit (Figure 1d–g), demonstrating its high adaptability and controllable response in a wide range of complex wound morphologies.

The device integrates a microcontroller unit (MCU) with Bluetooth Low Energy (BLE), which allows patients to precisely set and dynamically regulate the wound contraction strength through a mobile app (Figure 1h). This intelligent closure strategy allows for real‐time contraction adjustments based on personal comfort and avoids discomfort caused by over‐contraction, thus improving treatment accuracy, comfort, and compliance. In the initial state, the planar size of the MSWZ is approximately 30.34 × 29.88 mm, and the device is highly stretchable, superelastic, and flexible, recovering its initial morphology after extensive stretching, twisting, and bending, thus ensuring stability, fit, and comfort on the skin surface (Figure 1i). The fabrication process includes (Figure 1j): ① processing and encapsulation of Ni–Ti SMA mechanical metamaterials; ② preparation and transfer of PI/Cu conductive layer; and ③ patterning and skeletonization of Ecoflex elastomers.

### 2.2 Structural Optimization and Performance Characterization of MSWZ

**FIGURE 2:**
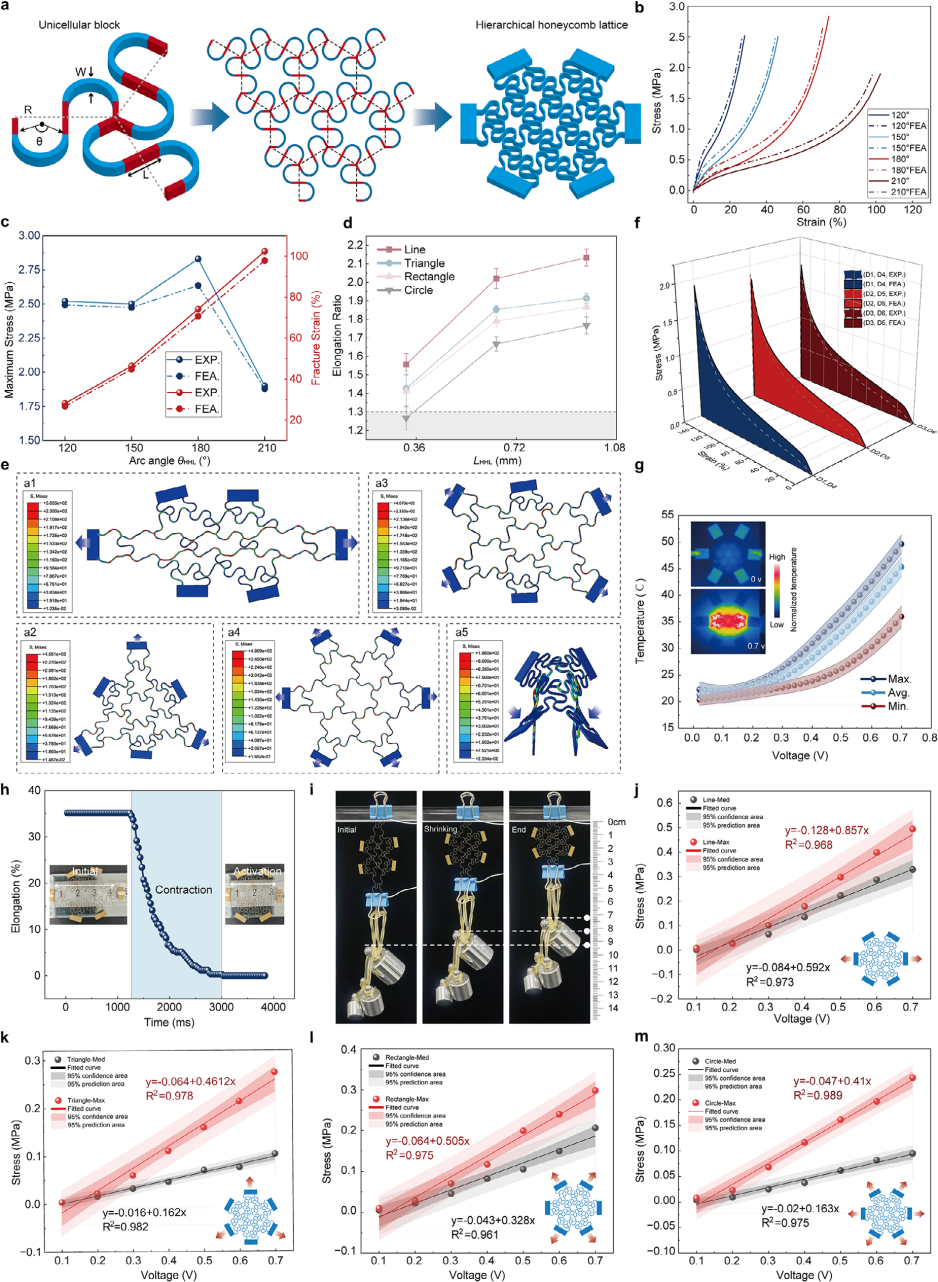
Structure optimization and performance characterization of MSWZ. (a) “Horseshoe” unicellular block and lattice topology strategy of HHL. (b) Stress–strain curves of HHL at different *θ*_HHL_. (c) Maximum stress and fracture strain of HHL at different *θ*_HHL_. (d) Correlation between residual elongation and *L*_HHL_ of HHL, N = 3. (e) FEA results of HHL under different wound conditions after tensile or bending loading: a1: Linear wound; a2: Triangular wound; a3: Rectangular wound; a4: Circular wound; a5: 180‐degree bending. (f) Mechanical isotropy of HHL. (g) Electro‐thermal coupling properties of HHL, N = 3, Max.: maximum temperature; Avg.: average temperature; Min: minimum temperature. (h) Electro‐mechanical response properties of HHL. (i) HHL lifting a 100 g weight. Electro‐programmable mechanical contraction of HHL under different morphological wound conditions: (j) linear wound; (k) triangular wound; (l) rectangular wound; (m) circular wound, Med: medium elongation and Max: maximum elongation.

Human skin exhibits a typical “J” shaped stress–strain response and contains natural strain‐limiting mechanisms to avoid excessive deformation and tissue damage [38]. Wound tissue retains the nonlinear mechanical properties characteristic of collagenous soft tissue, exhibiting a distinct “J”‐shaped stress–strain curve and spatially heterogeneous mechanical responses [39]. Local mechanical analysis reveals a stiffer wound core surrounded by softer regions, forming a characteristic “mechanical fingerprint” [40]. Consequently, under external loading, the wound and its surrounding areas undergo non‐uniform deformation [39]. This local mechanical mismatch may lead to strain or stress concentration, which in turn impairs the wound healing process [41]. Ideal mechanical dressings should therefore mimic the nonlinear mechanical characteristics of skin to reduce mechanical mismatch with wound tissue and minimize local strain/stress concentration, thereby providing a more favorable mechanical environment for the wound [42, 43]. To simulate such properties, the researchers constructed a bionic Ni–Ti SMA metamaterial based on a hierarchical honeycomb lattice (HHL) as the mechanical core of the MSWZ. Figure 2a illustrates the “horseshoe” unicellular block and lattice topology strategy of the HHL, in which the unicellular block consists of two identical circular arcs connected by a crossbar, whose main parameters include radius (*R*_HHL_ = 0.9 mm), width (*W*_HHL_ = 0.216 mm), thickness (*T*_HHL_ = 0.2 mm), arc angle (*θ*_HHL_), and crossbar (*L*_HHL_). Notably, the lattice can be freely stretched along six axial directions 60° apart, thus matching the morphology and scale of complex wounds. At low stretching levels, the HHL deforms mainly through bending; when stretched to high levels, the deformation mechanism shifts to tensile dominance. This mechanism shift leads to a rise in effective modulus with increasing strain, which in turn produces a skin‐like nonlinear mechanical response.

The researchers explored the critical parameters affecting the mechanical properties of HHL, i.e., arc angle (*θ*_HHL_) and crossbar (*L*_HHL_). Finite element analysis (FEA) and mechanical testing were used to carry out a systematic evaluation. First, *θ*_HHL_ was varied from 120° to 210° (in 30° steps) to explore the stress–strain curve characteristics of the HHL. The HHL exhibited “J” type stress–strain curves at *θ*_HHL_ = 120–210° (Figure 2b). Further analysis reveals that the fracture strain of HHL increases with increasing *θ*_HHL_, up to 102.41%, while the maximum stress of HHL peaks at 2.83 MPa at *θ*_HHL_ = 180° (Figure 2c and Figures S2 and S3). Considering that MSWZ needs to provide high mechanical output to counteract high skin tension while maintaining ductility, *θ*_HHL_ = 180° was finally chosen. To ensure effective contraction for different wound morphologies, the researchers pre‐stretched the HHL in different axial directions to the maximum level and then released it to measure the maximum residual elongation in each axial direction (Figure S4). The HHL corresponded to the linear, triangular, rectangular, and circular wound conditions, and the residual elongation was recorded as λElongation Line, λElongation Triangle, λElongation Rectangle, and λElongation Circle, respectively (Figure S5). Given the significant effect of *L*_HHL_ on overall extension, we examined *L*_HHL_ = 0.36, 0.72, and 1.08 mm. As shown in Figure 2d, residual elongation increased with increasing *L*_HHL_. Previous studies have indicated that wound contraction patches need to meet an extension of λ = 1.3 in the uniaxial direction for effective treatment [21].

The λElongation Line, λElongation Triangle, λElongation Rectangle, and λElongation Circle of the HHL at *L*_HHL_ = 0.72 mm were 2.02, 1.85, 1.79, and 1.67, respectively, all of which satisfy the therapeutic requirements. FEA further showed that at *L*_HHL_ = 0.72 mm, the internal distributed load was lower than the material tensile strength regardless of stretching according to different morphological wound conditions or after 180° bending, and the out‐of‐plane buckling of the structure remained insignificant, which verified the security margin and structural stability of the design (Figure 2e and Figure S6). The combination of size, ductility, and effectiveness resulted in *L*_HHL_ = 0.72 mm being adopted. To ensure the uniformity of wound edge shrinkage, the HHL was subjected to uniaxial mechanical testing along three horizontally spaced axial directions of 120° (D1, D4; D2, D5; D3, D6). As shown in Figure 2f, the overlap of the three stress–strain curves was 97.8%, which was in approximate agreement with FEA, indicating that the HHL has excellent isotropy.

The researchers further conducted a systematic study of the mechano‐electrical‐thermal coupling properties of HHL. Owing to the conductivity of Ni–Ti SMA and the continuous 2D conductive path of HHL, the warming with increasing voltage was visible under infrared thermography (Figure 2g and Figure S7). Considering that low‐temperature electromechanical shrinkage materials require low actuation temperatures (< 50°C) in bio‐integration applications, the safe voltage was limited to ≤ 0.7 V [22]. To investigate the electromechanical shrinkage performance of the HHL, the HHL was pre‐stretched along the ends and subsequently loaded with a 0.7 V electrical pulse, and the HHL accomplished a fast electromechanical shrinkage within 1.73 s (Figure 2h and Movie S1).

In operation, the energized HHL was capable of lifting a 100 g load—roughly 500 times its own mass—highlighting its substantial contractile strength and its potential for closing wounds subjected to high tensile stress (Figure 2i and Movie S2). To further assess its performance on wounds of varying geometries, the device was pre‐stretched according to the corresponding morphology and subsequently actuated within a voltage range of 0–0.7 V. Across all tested wound types, the mechanical output exhibited clear voltage‐dependent programmability, with strong linear correlations (R^2^ > 0.96). The contraction forces obtained under different configurations were as follows: linear wounds, 0–0.494 MPa (Figure 2j); triangular wounds, 0–0.274 MPa (Figure 2k); rectangular wounds, 0–0.298 MPa (Figure 2l); and circular wounds, 0–0.243 MPa (Figure 2m).

The optimized HHL ensures the mechanical properties of MSWZ. To evaluate the reliability and robustness of the MSWZ in a human daily‐wear environment, the researchers performed cyclic tensile tests on the MSWZ under both dry conditions and in an artificial sweat environment [44]. As shown in Figure S8a,b, whether in a dry state or after 48 h of immersion in artificial sweat, the MSWZ demonstrated stable stress output during 1,000 cycles of tensile testing (strain range: 5%–15%), proving the mechanical robustness of its structural design [45, 46]. Furthermore, as shown in Figure S8c,d, the MSWZ retains effective stretching and contracting capabilities even after cyclic testing under dry and artificial sweat‐soaked conditions. These results collectively indicate that the MSWZ possesses excellent mechanical robustness under daily human activity and physiological conditions.

These findings indicate that the HHL can deliver rapid and reliable multiaxial contraction forces that are adaptable to diverse wound morphologies. Moreover, its voltage‐controlled actuation enables fine‐tuning of the treatment intensity, allowing adjustments based on an individual patient's pain tolerance. HHL also ensures the robustness of MSWZ under daily human activity and physiological conditions. These properties support a more personalized wound management approach while enhancing patient comfort and overall treatment adherence.

### 2.3 Demonstration of the Practical Application of MSWZ

**FIGURE 3:**
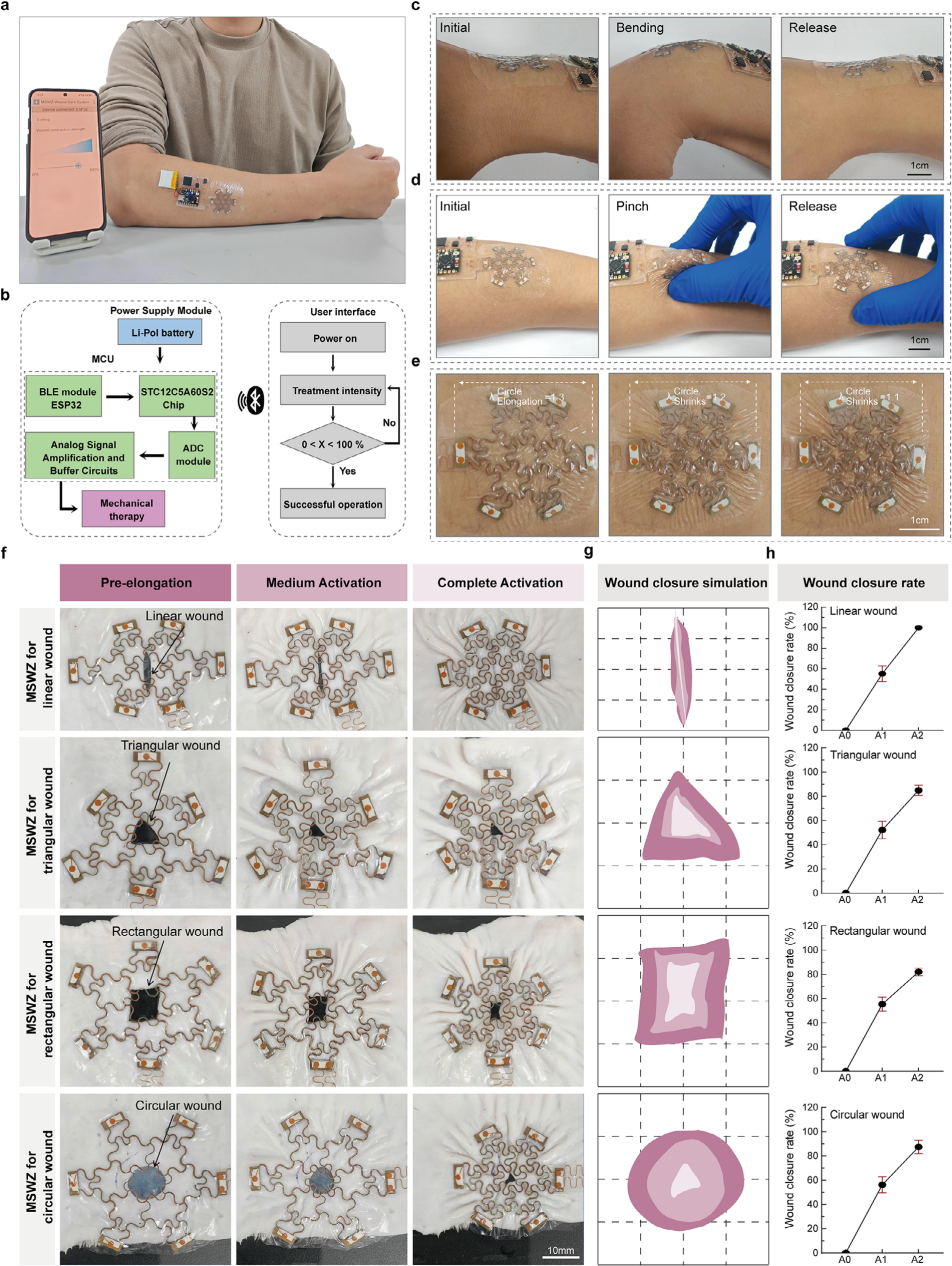
Application demonstration of MSWZ. (a) The flexible MCU of MSWZ integrates BLE communication and can be controlled by mobile terminal applications. (b) The core components of MSWZ and its manipulation logic. (c) MSWZ can work stably in high‐strain areas such as wrist joints. (d) The MSWZ maintains a seamless fit to the skin. (e) The MSWZ achieves programmable mechanical contraction against human skin tension. Validation of the programmable mechanical contraction of the MSWZ on rat skin wounds with different morphologies: (f) images illustrating mechanical contraction of the wounds by the MSWZ, (g) simulation of the wound‐closure process, and (h) variations in wound‐closure rates; A0: Initial state; A1: Medium activation; A2: Complete activation, N = 3.

To match the electrically programmable mechanical contraction of the HHL with the need for wearability, convenience, and precise personalized therapy, the researchers integrated a flexible MCU into the MSWZ, which employs a PDMS flexible substrate for conformal skin application and is powered by a 100 mAh, 3.7 V lithium soft‐packed battery. The MCU has a built‐in voltage modulation module that outputs electrical pulses of 0 to 0.7 V, and integrated BLE communication that allows patients to conveniently control the contraction intensity of the MSWZ via a user‐friendly mobile terminal application (Figure 3a). The system block diagram outlines the core components and their manipulation logic (Figure 3b).

The MSWZ remained adhered to the skin after being applied to the wrist joint and subjected to 90‐degree bending, demonstrating its reliable wearability on joints and other high‐strain areas (Figure 3c). When the MSWZ was applied to the forearm, it maintained a seamless fit to the skin, regardless of whether it was pinched or released (Figure 3d), ensuring that subsequent mechanical contraction could be effectively applied to the wound area. The researchers pre‐stretched the MSWZ based on circular wound conditions and then applied it to the skin of the forearm. The programmable features of the MSWZ were validated by controlling the MCU via a cell phone application, which allowed for incremental increases in mechanical contraction (Figure 3e).

To demonstrate programmable mechanical contraction for different wound morphologies, MSWZ was used for evaluation in free rat skin wounds. Maximum pre‐stretch was applied according to the linear, triangular, rectangular, and circular wound conditions, yielding λElongation Line, λElongation Triangle, λElongation Rectangle, and λElongation Circle of 2.02, 1.85, 1.79, and 1.67, respectively, which were followed by the application of MSWZ. MSWZ was applied and activated incrementally, and the wound closure rate was calculated. The results showed (Figure 3f,g and Movie S3) that the MSWZ could achieve complete closure for linear wounds without skin defects, while for the rest of the wounds with different morphologies, a significant incremental closure was observed. The MSWZ achieved the maximum wound closure rates of 0–‐100%, 0–84.85%, 0–81.99%, and 0–87.4% for linear, triangular, rectangular, and circular wounds, respectively (Figure 3h). In summary, the MSWZ demonstrated excellent performance in terms of flexible wearability, voltage programmable control, and polymorphic wound adaptability. The integrated flexible MCU significantly improves patient autonomy, not only enhancing treatment comfort and compliance but also enabling quantifiable control of the wound mechanical contraction process. Together, these features ensure precise treatment.

### 2.4 Biocompatibility Validation of MSWZ

**FIGURE 4:**
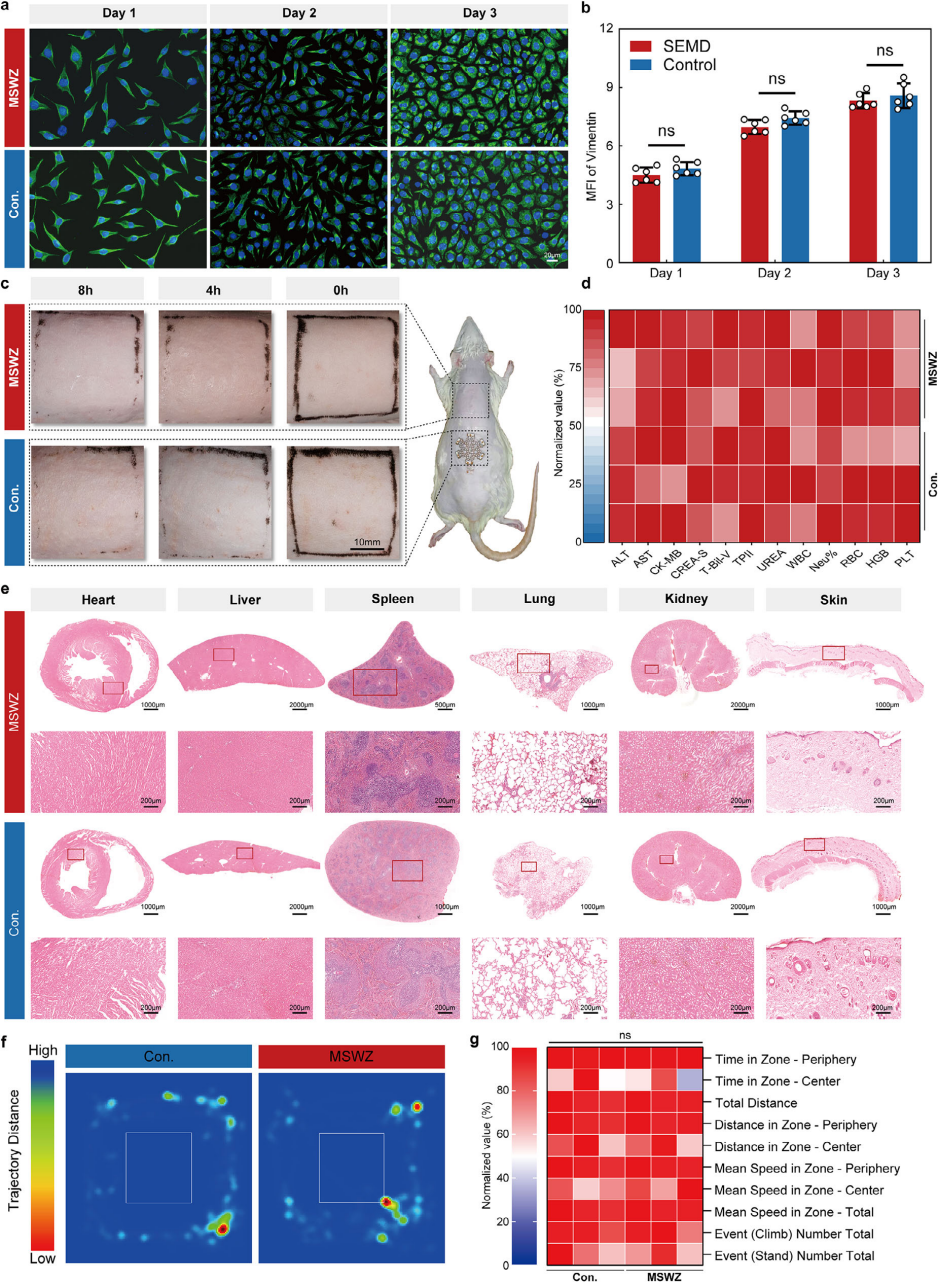
Biocompatibility validation of MSWZ. (a) Fluorescence images of cell morphology and proliferation. (b) Comparison of cell density and proliferation viability, N = 6. (c) Rat skin stimulation experiment. (d) Analysis of whole blood and serum biochemical detection indexes of rat venous blood, N = 3. (e) H&E staining results of rat organs. (f) Thermogram of rat activity trajectory. (g) Behavioral indicators of rats, N = 3. Data are presented as mean ± SD. Two‑way ANOVA with Tukey's post hoc test or a two‑tailed Student's t‑test was used; ns: no statistically significant.

The biocompatibility of MSWZ must be systematically evaluated before treatment. To this end, the researchers performed a combination of in vitro cytology experiments and in vivo animal experiments to evaluate the biocompatibility of MSWZ. The mouse fibroblasts (NCTC clone 929, L929) were co‐cultured with MSWZ extracts, and a control group of standard culture was set up for three days. Cell morphology and proliferation were observed by immunofluorescence (IF) labeling of Vimentin. The results of laser confocal microscopy assay showed that at day 1, both the MSWZ group and the control group showed the morphology of adherent‐growing spindle‐shaped cells; during the following two days, the cells in both groups gradually proliferated and formed clusters of cells with similar density and morphology (Figure 4a). Quantitative analysis by mean fluorescence intensity (MFI) revealed that there was no significant difference in cell density and relative proliferation vigor between the MSWZ group and the control group (Figure 4b). These results indicated that MSWZ exhibited good cytocompatibility.

Systematic in vivo evaluations were performed, including skin irritation test, histopathological analysis, and blood biochemical tests. In the skin irritation test, MSWZ was applied to the back of a dehairing SD rat, and a medical PU dressing was used as a control. Within 8 h, neither group exhibited irritation reactions such as blisters, redness, or rashes, indicating that MSWZ is mild and non‐irritating to the skin (Figure 4c). The skin was sampled and analyzed by hematoxylin‐eosin (H&E) staining. The MSWZ was implanted subcutaneously into SD rats to further evaluate systemic biosafety, with a sham‐operated group serving as the control. Heart, liver, spleen, lung, kidney, and venous blood samples were collected from both groups of rats after two weeks. Major organs underwent histological evaluation by H&E staining, while biochemical analysis of venous whole blood and serum was performed by venous blood samples. Results showed no significant difference between the MSWZ group and the control group in the following functional indicators: cardiac function: creatine kinase isoenzyme type MB (CK‐MB); hematopoietic function: red blood cell count (RBC), hemoglobin (HGB), and platelet count (PLT); hepatic function: alanine aminotransferase (ALT), aspartate aminotransferase (AST), total bilirubin (T Bil‐V), total protein (TPII); renal function: serum creatinine (CREA‐S), urea (UREA); immune function: white blood cell count (WBC), neutrophil percentage (Neu%) (Figure 4d). The H&E staining results confirmed that no pathological degeneration, necrosis, or inflammatory cell infiltration was observed in all organs, indicating that MSWZ implantation did not induce systemic toxicity or organ damage (Figure 4e).

Given the critical impact of wearing comfort on patient compliance, the open field test (OFT) was performed to evaluate whether the MSWZ triggers pain or stress [31]. Rats wearing the MSWZ were compared to control rats with only a PU dressing. Trajectory thermograms validated the consistency of behavioral patterns between the two groups of rats (Figure 4f). In addition, rats wearing the MSWZ were not significantly different from the control group in all behavioral indices (activity distance, speed, center dwell time, etc.) (Figure 4g). This indicates that wearing the MSWZ does not cause discomfort or stressful behavior. Combining the results of in vitro and in vivo experiments, MSWZ showed excellent performance in terms of cytocompatibility, skin gentleness, systemic biosafety, and wearable comfort, which demonstrated its excellent biosafety and potential for clinical application, and provided the conditions for subsequent validation of wound repair.

### 2.5 Validation of MSWZ for the Repair of Linear Wounds in Rats

**FIGURE 5:**
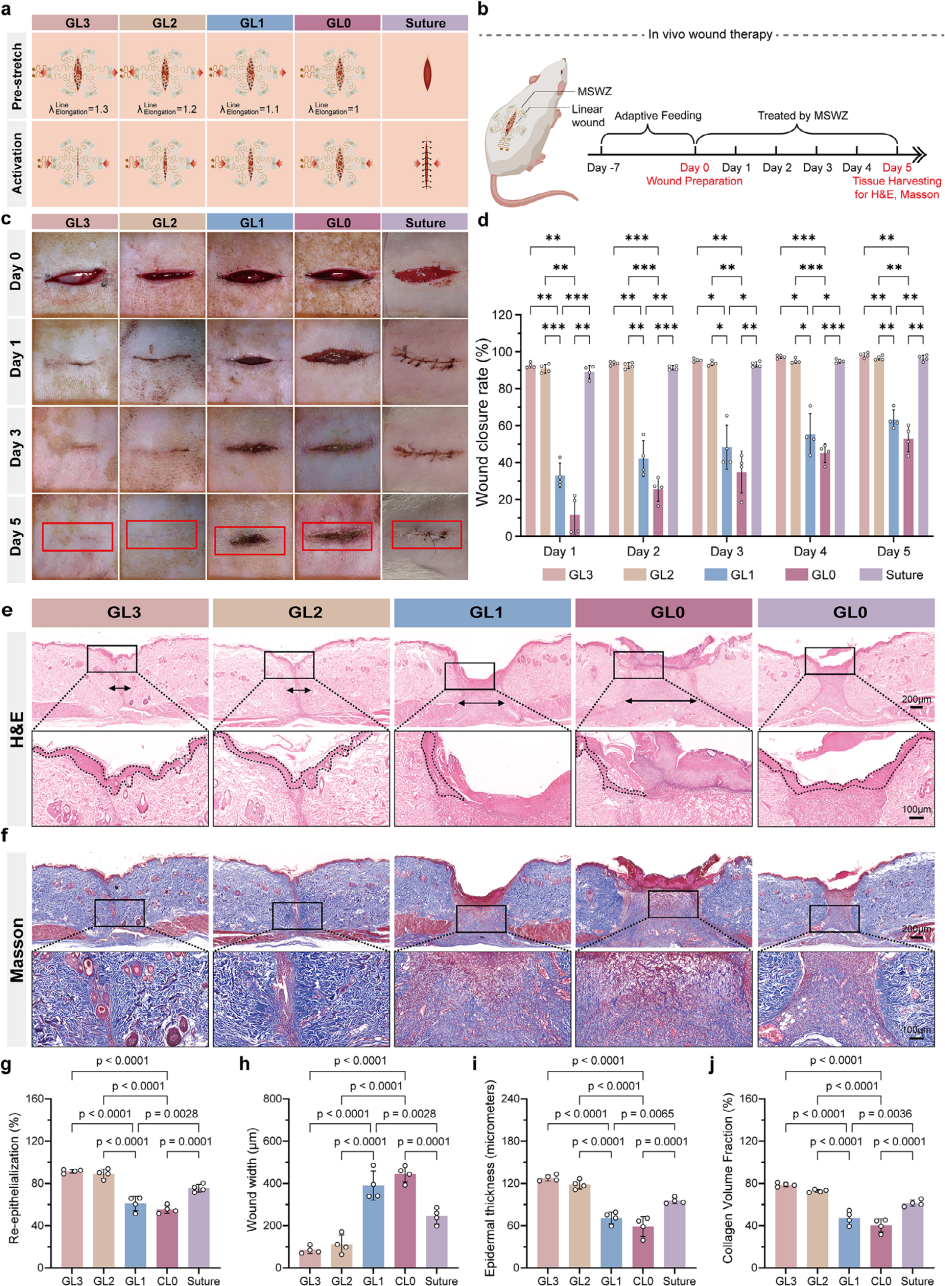
Validation of MSWZ for linear wound repair in rats. (a) Mechanical contraction of MSWZ with different pre‐stretching levels for the linear wound condition. (b) Timeline of animal experiments. (c) The healing images of linear wounds. (d) Histogram of wound closure rate and comparative analysis. (e) H&E staining images of wounds. (f) Masson staining images of wounds. Statistical analysis of histological indices of the wound: (g) re‐epithelialization, (h) wound width, (i) epidermal thickness, and (j) collagen volume fraction. Data are presented as mean ± SD. One‐way or two‐way ANOVA with Tukey's post hoc test was performed. Sample size N = 4. *p < 0.05, **p < 0.01, ***p < 0.001. P < 0.05 was considered statistically significant.

To validate the efficacy of MSWZ in the treatment of linear wounds without skin defects, the researchers used a scalpel to create a linear wound of approximately 10 mm in length (deep to the superficial fascia) on the back of SD rats. The MSWZ was then applied to the wound and activated. According to the degree of pre‐stretching (for the linear wound condition), there were five groups: λElongation Line = 1.3, defined as group GL3; λElongation Line = 1.2 as group GL2, λElongation Line = 1.1 as group GL1; a control without pre‐stretching defined as group GL0, and a group using surgical suturing is defined as the Suture group (Figure 5a).

The MSWZ was changed every 1 day, and the wound was cleaned and photographed; wound tissue was harvested for further histopathological analysis at the end of the experiment (Figure 5b). As shown in Figure 5c, at day 1, the wounds in the GL3, GL2 and Suture groups had reached near‐complete closure; in contrast, the GL1 and GL0 groups were still in an open state. This indicates that at λElongation Line = 1.2, MSWZ can achieve immediate closure of linear wounds, which is important for emergency treatment. By day 5, the wounds in the GL3 and GL2 groups were no longer obvious, and no scabs were seen around the wound margins, suggesting that the epidermal barrier was effectively restored; Suture group wounds had closed, but the wound lines remained more prominent than the CL3 and CL2 groups, and a slight scab was still visible; this may be due to knot reaction or inappropriate suturing tension [41]; Whereas the wounds in the GL1 and GL0 groups were still clearly visible, and scabs were present on the surface, suggesting that reepithelialization was lagging and accompanied by granulation tissue exposure. Combined with the analysis of the wound closure rate in Figures 5d, it can be seen that the difference between the GL3 group (98.06%), GL2 group (96.64%) and Suture group (96.47%) was not significant, and both of them were significantly better than the GL1 group (63.22%) and GL0 group (52.96%).

Further H&E and Masson staining was performed, as shown in Figure 5e, the width of the wound was significantly narrowed in the GL3 and GL2 groups, and the neocutaneous structure was clear; the formation of the keratinized layer was complete, and the heckle cells were arranged densely and regularly [47]. Despite the satisfactory epidermal regeneration in the Suture group, the wound width remained greater than the GL3 and CL2 groups. In contrast, the healing process was poorer in the GL1 and GL0 groups: the wound was still wider, and the new epidermis at the margins did not completely cover the wound, resulting in the exposure of tender granulation. As shown in Figure 5f, the GL3 and GL2 groups showed significant collagen fiber deposition within the wound. These fibers were densely and regularly arranged, resembling the surrounding normal skin, and overall superior to the Suture group. In contrast, the GL1 and GL0 groups exhibited only sparse collagen fibers with a loose and disorganized structure, suggesting a poor tissue remodeling. Based on the results of H&E and Masson, further quantification was performed (Figures 5g–j): the re‐epithelialization rates in the GL3 group (91.46%) and the GL2 group (88.90%) were significantly higher than the Suture group (75.47%), and were also significantly higher than the GL1 group (61.02%) and the GL0 group (55.53%). The wound widths in the GL3 and GL2 group were significantly narrower than the Suture group, GL1 group, and GL0 group. The thickness of the neoepidermis in the GL3 Group and GL2 Group was significantly higher than the Suture Group, GL1 Group, and GL0 Group. Collagen volume fraction: The GL3 group (78.71%) and GL2 group (72.92%) were significantly higher than the Suture group (61.24%), GL1 group (47.33%), and GL0 group (40.28%).

Further analysis of wound closure speed shows that the MSWZ completely closed the wound in approximately 4 s, whereas a skilled surgeon required approximately 2 min and 47 s (Movie S4). This highlights the MSWZ's ability to enable rapid treatment in emergency situations. These results indicate that in the linear wound model, MSWZ at λElongation Line = 1.3 or 1.2 can accelerated re‐epithelialization, significantly promoting collagen deposition and tissue remodeling. Moreover, MSWZ outperforms surgical suturing in both healing outcomes and wound closure speed.

### 2.6 Validation of MSWZ for the Repair of Circular Wounds in Rats

**FIGURE 6:**
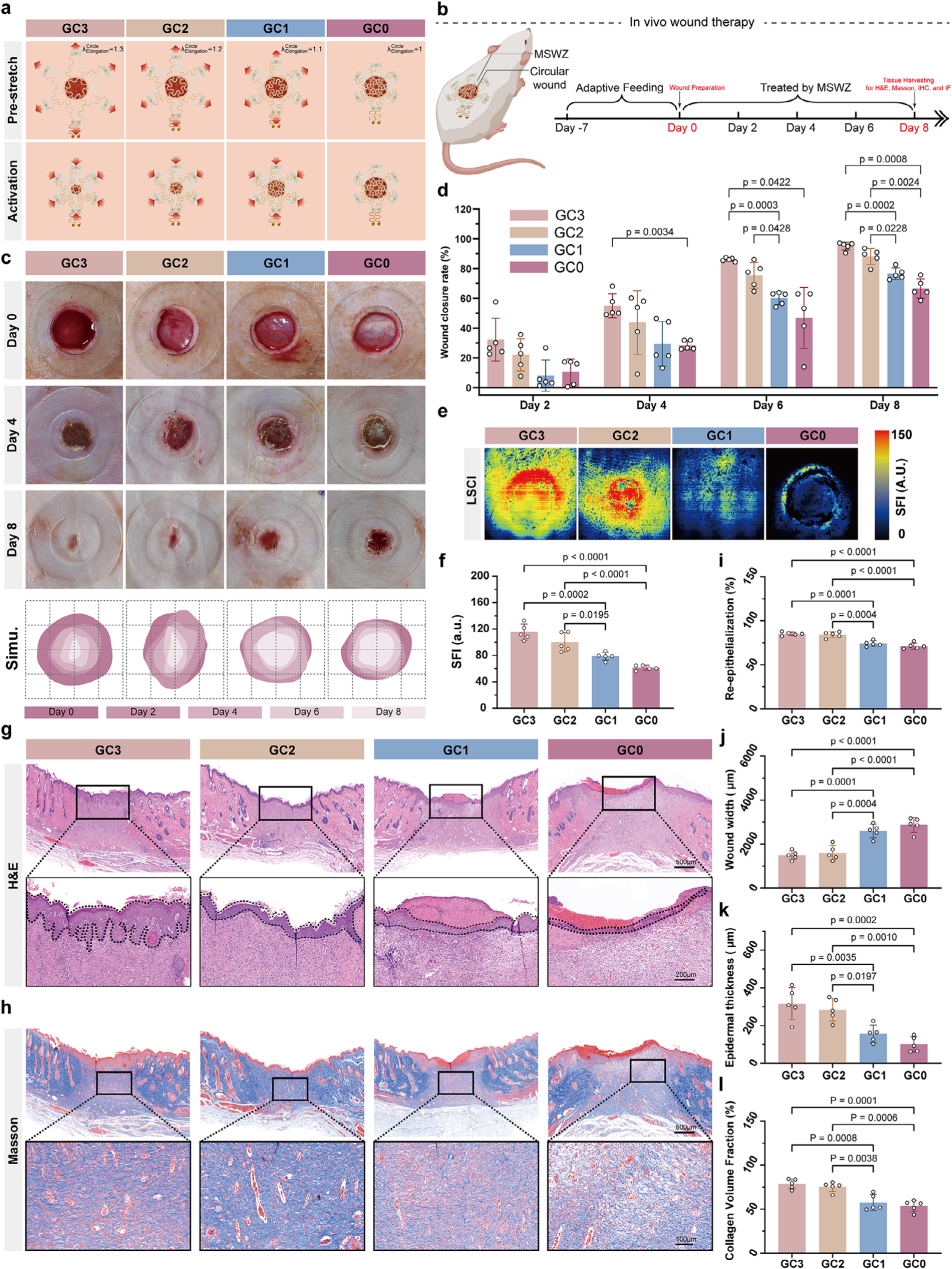
Validation of MSWZ for the repair of circular wounds in rats. (a) Mechanical contraction of MSWZ with different pre‐stretching levels for circular wound condition. (b) Timeline of animal experiments. (c) Healing images of linear wounds. (d) Histogram of wound closure rate and comparative analysis. (e) LSCI of wound perfusion. (f) Quantitative analysis of wound perfusion, a.u.: arbitrary unit. (g) H&E staining images of wounds. (h) Masson staining images of wounds. Statistical analysis of histological indices of the wound: (i) re‐epithelialization, j) wound width, (k) epidermal thickness, and (l) collagen volume fraction. Data are presented as mean ± SD. One‐way or two‐way ANOVA with Tukey's post hoc test was performed. Sample size N = 5; p < 0.05 was considered statistically significant.

To further investigate the therapeutic effect of MSWZ on skin defect wounds, the researchers created circular wounds with a diameter of 10 mm (deep to the superficial fascial layer) on the back of SD rats for validation. According to the degree of pre‐stretching (for the circular wound condition), the rats were sequentially categorized into the following groups: λElongation Circle = 1.3 for group GC3, λElongation Circle = 1.2 for group GC2, λElongation Circle = 1.1 for group GC1, and the control with only MSWZ applied for group GC0 (Figure 6a). The MSWZ was changed every 2 days, the wounds were cleaned and photographed, and the wound tissues were harvested for further histopathological analysis at the end of the experiment (Figure 6b).

As shown in Figure 6c, by day 8, the wounds in the GC3 and GC2 groups had healed significantly without superficial scabbing, indicating effective restoration of the epithelial barrier. In contrast, wounds in the GC1 group were more distinct, with tender granulation tissue visible within the wound; whereas wounds in the GC0 group were equally distinct, with surface scabbing confirming delayed epithelial regeneration. Wound closure rates were further analyzed and were significantly better in the GC3 and GC2 groups at 95.07% and 88.11%, respectively, than in the GC1 group (76.49%) and the GC0 group (69.95%) (Figure 6d). In addition, the researchers further assessed the variation of blood perfusion during the wound healing process by Laser Speckle Contrast Imaging (LSCI) of day 4 wounds. The results suggested that the blood flow signals in the GC3 and GC2 groups were superior to those in the GC1 and GC0 groups, where the blood flow perfusion in the GC3 group was elevated by 81.53% relative to that in the GC0 group (Figure 6e,f). This indicated that the mechanical contraction of MSWZ could effectively promote the recovery of wound perfusion.

Histological assessment was performed on the rat wound tissues. H&E staining showed that the newly formed epidermis in the GC3 and GC2 groups had fully covered the wound bed, and the stratified structure of mature epidermal tissue was clearly identifiable. In contrast, specimens from the GC1 and GC0 groups displayed an immature epidermal layer that failed to establish an effective epithelial barrier, accompanied by noticeable inflammatory exudate on the wound surface (Figure 6g). Masson staining further demonstrated that collagen fibers in the GC3 and GC2 groups were densely packed and arranged in an orderly pattern, reflecting more advanced matrix remodeling. By comparison, collagen in the GC1 and GC0 groups appeared loosely distributed with irregular orientation (Figure 6h). Quantitative analyses of these histological results are summarized in Figure 6i–l. As shown in Figure 6i, the re‐epithelialization rates in the GC3 and GC2 groups reached 85.09% and 84.02%, respectively, which were markedly higher than those observed in GC1 (74.05%) and GC0 (71.28%). Consistent with this, wound width was significantly reduced in the GC3 and GC2 groups compared with the other two groups (Figure 6j). The regenerated epidermis was also substantially thicker in GC3 and GC2 than in GC1 and GC0 (Figure 6k). Moreover, the collagen volume fraction reached 78.74% and 75.34% in GC3 and GC2, respectively, whereas GC1 and GC0 showed notably lower values of 57.50% and 53.68% (Figure 6l). Collectively, these findings indicate that the MSWZ markedly enhances epidermal regeneration and collagen matrix reconstruction in the circular skin defect model.

### 2.7 Validation of MSWZ in Repairing Clinical Wound Morphology

Previous studies have shown that the most common wound morphologies in clinical practice include spindle‐shaped and oval‐shaped wounds [48, 49, 50, 51]. Therefore, the researchers constructed wounds with similar morphologies on the backs of SD rats, referencing two clinical cases of spindle‐shaped and oval‐shaped wounds reported in the literature [21]. Subsequently, an experienced surgeon adjusted the pre‐stretching direction and magnitude of the MSWZ based on the wound morphology, and applied the MSWZ for wound treatment, while setting untreated wounds as the control group.

As shown in Figure S9a, pre‐stretched MSWZ was applied to the back of SD rats to induce contraction and closure of a spindle‐shaped wound (wound dimensions: 15 mm × 9 mm). By day 2, the MSWZ‐treated wound had significantly shrunk, while the wound in the blank control group had further expanded, likely due to skin surface tension pulling; by day 14, the MSWZ‐treated wound had completely closed with no visible scab on the surface, whereas the wound in the blank control group remained clearly visible, with fresh scabs on the wound surface indicating that the epidermal barrier had not yet recovered (Figure S9b). As shown in Figure S9c, the wound closure rate in the MSWZ group was significantly superior to that of the blank control group starting from Day 2; by Day 10, the wound closure rate in the MSWZ group had exceeded 90%. By day 14, the wound closure rate in the MSWZ group was 97.25%, significantly higher than the blank control group (79.20%). This suggests that MSWZ is compatible with spindle‐shaped wounds, not only providing mechanical protection to prevent wound enlargement due to skin tension but also accelerating wound closure.

As shown in Figure S10a, pre‐stretched MSWZ was applied to the backs of SD rats to induce the contraction and closure of an oval‐shaped wound (dimensions: 15 mm × 12 mm). By Day 2, the wound treated with MSWZ had significantly shrunk, while the wound in the blank control group had further expanded; By day 14, the MSWZ‐treated wound had completely closed with no visible scab on the surface; the wound in the blank control group remained clearly visible, and the fresh scab on the wound surface indicated that the epidermal barrier had not yet recovered (Figure S10b). As shown in Figure S10c, the wound closure rate in the MSWZ group was significantly higher than the blank control group starting from day 2; By day 14, the wound closure rate in the MSWZ group was 94.02%, significantly higher than the blank control group (70.66%). This suggests that MSWZ is also compatible with oval‐shaped wounds, providing mechanical protection while accelerating wound closure.

### 2.8 Mechanisms by Which MSWZ Promotes Wound Repair

**FIGURE 7:**
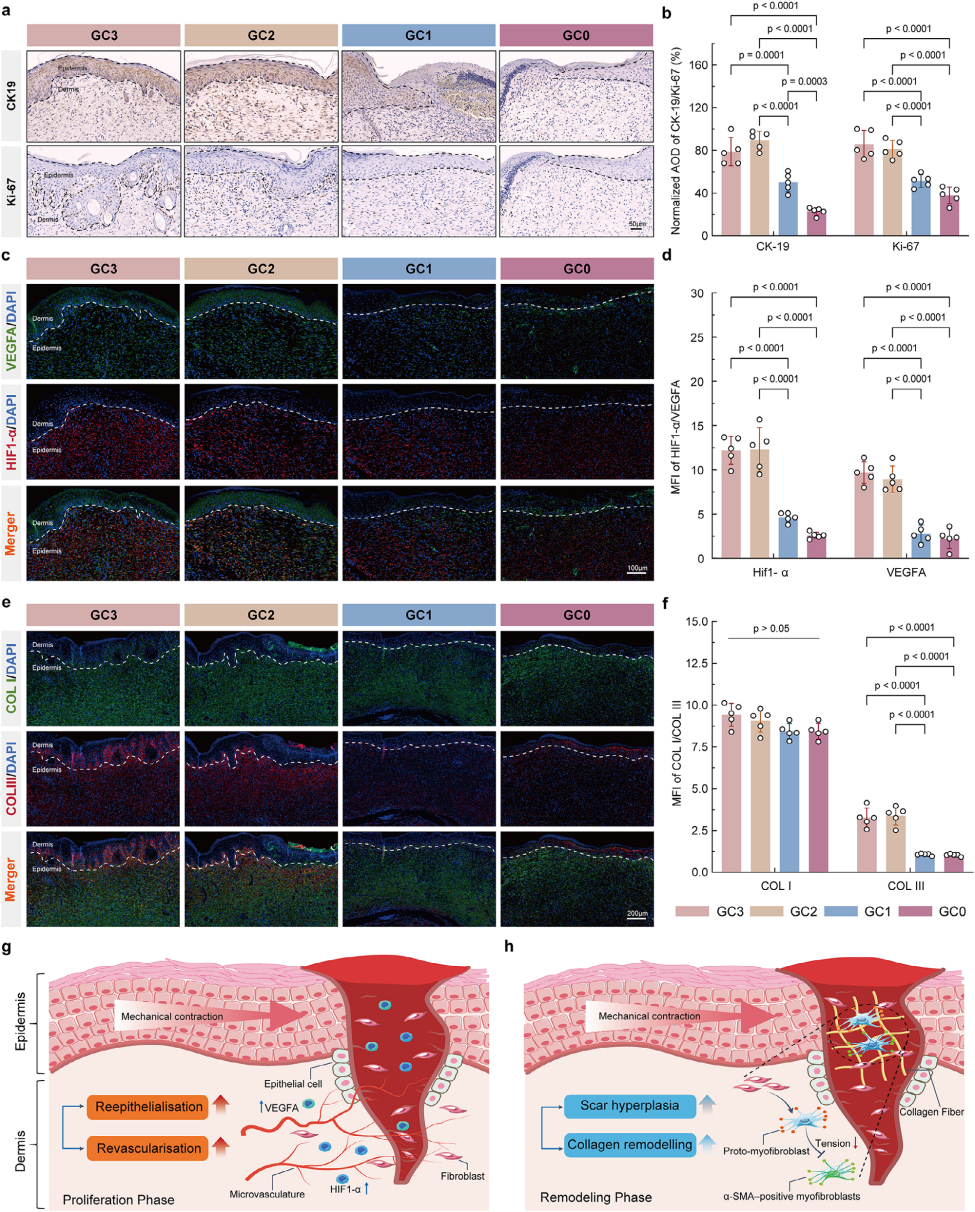
Mechanism of MSWZ promoting wound repair. (a) IHC images of Ki‐67 and CK19 in wound tissues. (b) Quantitative analysis of Ki‐67 and CK19 between groups, AOD: Average Optical Density. (c) IF images of HIF‐1α and VEGFA in wound tissues. (d) Quantitative analysis of HIF‐1α and VEGFA between groups. (e) IF images of Col I and Col III. (f) Quantitative analysis of Col I and Col III between groups. (g) Mechanism diagram of MSWZ promoting re‐vascularization and re‐epithelialization. (h) Mechanism diagram of MSWZ improving wound matrix remodeling. Data are presented as mean ± SD. One‐way ANOVA with Tukey's post hoc test was performed. Sample size N = 5. P < 0.05 was considered statistically significant.

The researchers examined the keratinocyte marker CK19 and the cell proliferation marker Ki‐67 to elucidate the mechanism by which MSWZ promotes wound repair. Immunohistochemistry (IHC) revealed that the expression of CK19 in the epidermal layer was intense and regularly distributed in the GC3 and GC2 groups, while the expression of CK19 in the GC1 and GC0 groups was weak, in contrast, suggesting that the process of re‐epithelialization was lagging (Figure 7a,b). The densities of Ki‐67‐positive cells in the epidermal layer of the GC3 and GC2 groups were 755 and 714 cells/mm^2^, respectively, which were significantly higher than those of the GC1 group (453 cells/mm^2^) and the GC0 group (334 cells/mm^2^), suggesting that MSWZ promotes the proliferation and migration of keratinocytes, thus accelerating re‐epithelialization (Figure 7a,b). The vascular regeneration during the proliferative phase of wound repair is closely associated with epithelial regeneration [3, 52]. The vascular endothelial marker CD31 was further examined to assess the impact of MSWZ on revascularization. The IHC results showed higher microvessel densities in the GC3 and GC2 groups, 283 and 257/mm^2^, respectively, which were significantly higher than those in the GC1 group (153/mm^2^) and the GC0 group (101/mm^2^) (Figure S11). These results indicated that MSWZ enhanced the microangiogenesis of the wound and provided oxygen and nutrient support for the migration and proliferation of epithelial cells.

During the early stages of wound formation, localized relative hypoxia stabilizes hypoxia‐inducible factor‐1α (HIF‐1α) and promotes its nuclear translocation. This subsequently upregulates the transcription of a series of angiogenesis genes, including vascular endothelial growth factor A (VEGFA), thereby driving endothelial cell proliferation, migration, and neovascularization [53, 54]. Remarkably, exogenous mechanical stress could further stabilize HIF‐1α expression through mechanosensitive pathways [55]. IF results revealed that HIF‐1α and VEGFA fluorescence signals were significantly enhanced in the GC3 and GC2 groups, and both of their MFI were higher than the GC1 and GC0 groups (Figure 7c,d). To confirm that the above results correlate with mechanosensitive pathway activation, Yes‐Associated Protein (YAP) and Vimentin IF staining was performed. Vimentin labels interstitial cells in the wound, while YAP, as a classic mechanosensitive signaling molecule, reflects the cellular response to exogenous mechanical stimuli and is involved in the regulation of angiogenesis [56, 57]. The results revealed that the GC2 and GC3 groups exhibited significantly higher mean YAP fluorescence intensities within DAPI‐stained nuclear regions and within Vimentin‐positive regions compared to the GC0 and GC1 groups, indicating that mechanical contraction of the MSWZ enhances the accumulation of YAP in nuclear‐associated regions and Vimentin‐positive stromal regions (Figures S12). These results reveal that MSWZ may activate the mechanosensitive pathway, upregulate angiogenesis‐related factors, and thereby accelerate angiogenesis and perfusion restoration, ultimately promoting epithelial repair.

The wound remodeling phase plays a decisive role in determining the final quality of tissue repair [3]. During this stage, fibroblasts synthesize new collagen while simultaneously regulating the turnover of pre‐existing fibers, shifting the wound matrix from a provisional scaffold toward a more stable and functional structure [58, 59]. To evaluate this remodeling process, type I collagen (Col I) and type III collagen (Col III) were analyzed. IF showed that Col I levels were comparable across all groups, whereas Col III expression was markedly higher in the GC3 and GC2 groups than in GC1 and GC0 (Figure 7e,f). As a consequence, the Col I/Col III ratios in the GC3 and GC2 groups were 2.94 and 2.76, respectively—substantially lower than the values observed in GC1 (7.97) and GC0 (8.26) (Figure 7f and Figure S13). A reduced Col I/Col III ratio is generally associated with a remodeling trajectory closer to physiological healing and is indicative of a lower likelihood of pathological scar formation [60].

To further examine fibroblast activity, the researchers assessed the expression of α‐SMA, a marker of activated myofibroblasts. α‐SMA levels were significantly lower in the GC3 and GC2 groups compared with the GC1 and GC0 groups (Figure S14), suggesting that MSWZ reduced mechanical tension at the wound edge and thereby limited excessive myofibroblast activation. These observations support the conclusion that MSWZ positively modulates collagen matrix organization by mitigating margin tension and promoting more orderly remodeling [61].

Overall, MSWZ promotes revascularization during the proliferation phase via HIF‐1α/VEGFA, providing energy and nutrients for re‐epithelialization (Figure 7g). Furthermore, during the remodeling phase, MSWZ inhibits excessive myofibroblast activation by alleviating wound margin tension, thereby improving wound matrix remodeling and reducing scarring risk (Figure 7h).

### 2.9 Performance Comparison and Potential Functional Extensions of MSWZ

We conducted a comprehensive comparison of MSWZ with reported wound‐closing dressing systems, performing a thorough comparison and analysis across key dimensions such as material type, activation method, response time, contractile force, adaptability to wound morphology, and complexity of the loading mechanism (Table S1 and Figure S15).

Given that human skin temperature and humidity are significantly influenced by environmental factors, mechanical contraction triggered by body temperature, moisture, or warm water may be unstable or unpredictable in practical applications [9, 21, 22, 23]. The MSWZ employs an electrothermal drive mechanism, offering greater stability, and enables electronically programmable mechanical contraction.

Despite not achieving the highest maximum contraction force, the MSWZ's performance significantly outperforms the vast majority of reported systems. More importantly, by leveraging the BLE module integrated with the MCU, the MSWZ enables real‐time, electronically programmable contraction, allowing users to dynamically adjust the contraction intensity based on their pain tolerance and clinical needs. For example, diabetic patients often suffer from peripheral neuropathy, resulting in reduced pain perception, while others may be more sensitive to pain [24]. Furthermore, traditional suturing or closure methods rely heavily on the operator's experience, making it difficult to quantitatively control closure tension [62]; the programmable contraction of the MSWZ enables quantitative output of closure force, ensuring treatment consistency while prioritizing patient comfort.

The MSWZ features the fastest contraction response time and is capable of rapid contraction when driven by low voltage (≤0.7 V). Combined with its user‐friendly interface, it enables rapid and effective wound closure even in emergency situations where medical professionals lack. Additionally, MSWZ supports various wound morphologies, fully addressing the diverse and complex wound morphology requirements in clinical practice [16]. Notably, MSWZ is reusable, and its pre‐stretched reloading method at room temperature is simple to operate, offering a distinct advantage over comparable studies. This advantage significantly reduces operational complexity and potential medical costs, enhancing the feasibility of practical application.

The MSWZ serves as an excellent dressing platform capable of integrating multifunctional components tailored to the treatment needs of various chronic wounds. As shown in Figure S16, by integrating a biocompatible, stretchable Mo electrode at the base of the MSWZ—featuring a stretchable design that conforms to the MSWZ's Ni‐Ti SMA metamaterial and is continuously powered by the MSWZ's MCU [32]—it is possible to specifically amplify the attenuated endogenous electric field in chronic wounds, thereby achieving electromechanical synergistic intervention to promote wound healing [63]. When addressing infected wounds, encapsulating a thin layer of antimicrobial hydrogel coating at the base of the stretchable electrode can provide additional antimicrobial properties [64].

Overall, MSWZ outperforms existing wound closure dressing systems and traditional closure techniques in terms of programmable contraction, response speed, morphological adaptability, and reusability, offering a new solution for personalized, precision wound treatment. Furthermore, MSWZ serves as an excellent dressing platform with multifunctional expansion capabilities, demonstrating potential for the treatment of complex chronic wounds.

## 3 Conclusion

In this work, the MSWZ integrates Ni/Ti‐SMA‐based mechanical metamaterials with stretchable electronic circuits, enabling a rapid actuation response within a few seconds under low driving voltages (≤0.7 V). The structural design permits multi‐axis stretching along six uniformly distributed directions, allowing the device to conform to wounds of varying morphologies and dimensions. Through a flexible MCU interface, the MSWZ provides adjustable mechanical contraction forces—0–0.494, 0–0.274, 0–0.298, and 0–0.243 MPa for linear, triangular, rectangular, and circular wounds, respectively—and achieves corresponding wound closure rates of 0–100%, 0–84.85%, 0–81.99%, and 0–87.4%. In animal models, the MSWZ produced immediate closure of linear wounds without tissue loss and improved the healing of circular wounds by 35.91%. In the two typical wound morphologies reported in clinical cases (spindle‐shaped and oval‐shaped wounds), MSWZ also demonstrated effective mechanical protection and accelerated wound healing.

Mechanistically, the device enhances both revascularization and re‐epithelialization, likely through stabilization of HIF‐1α and the subsequent upregulation of VEGFA. It also modulates matrix remodeling by reducing excessive myofibroblast activation, an effect attributed to its ability to lower tension at the wound margin, thereby contributing to improved tissue organization and a reduced risk of scarring. Collectively, these findings indicate that the MSWZ can deliver rapid, robust, and programmable multi‐axis mechanical contraction suited for personalized management of diverse wound types. Its conformability to skin and favorable safety profile further support its potential for future clinical translation.

## 4 Experimental Section

### 4.1 MSWZ Preparation and Process

Ni–Ti SMA sheets (200 µm thickness) were fabricated into HHL structures at a UV nanosecond laser platform (DL501U, Dezhong). The HHL was then immersed in Ecoflex (00‐20, Smooth‐On) and transferred to a glass substrate sprayed with a release agent (710, Wocax) and dried in a vacuum oven (AX30, Carbolite) at 45°C for 5 min. After curing of the Ecoflex, through‐holes were machined in the HHL ports for connection to the PI/Cu circuits at the same laser platform. The six circuit connections of the HHL were aligned with pre‐made PI masks, scraped with conductive silver paste, and dried in a vacuum oven at 45°C for 5 min. The PI/Cu foil (50 µm thick) was applied with a hydrosol, and the serpentine circuit pattern was eroded on the laser platform. The patterned PI/Cu was transferred to the six circuit interfaces of the HHL, and the hydrosol was removed with deionized water. On the surface of the PI/Cu‐coated HHL, a layer of Ecoflex was spin‐coated as an encapsulation layer by a spin‐coater (KW‐4B, Beijing Sedex Electronics Co., Ltd.), and dried in a vacuum oven at 45°C for 60 min. Subsequently, the encapsulated HHL was placed on the laser platform and skeletonized along the predefined lattice path. Before use, the HHL was sandwiched between two layers of PU dressing (1622 W, 3 M). A circular notch with a radius of approximately 10 mm was made in the center of the bottom PU layer to ensure that the device adhered only to the wound margins and not to the center of the wound, thus avoiding mechanical contraction forces acting directly on the central tissue.

### 4.2 Control Components

The flexible MCU is based on PDMS (Sylgard 184, Dow Corning) and integrates three types of core modules: (i) a power conversion unit with voltage regulation; (ii) a lightweight, soft‐packed lithium polymer battery (3.7 V / 100 mAh); and (iii) a BLE wireless communication module compliant with the Bluetooth 5.0 protocol. The system is accompanied by a mobile application that allows the operator to remotely control the mechanical shrinking process of the device through the BLE module.

### 4.3 Electro‐Thermal‐Mechanical Performance Testing

#### 4.3.1 Stress–Strain Test

Install HHL in a horizontal tensile tester (IBTC300s, Kelvin Measurement & Control System Co., Ltd.) to perform stress‐strain test.

#### 4.3.2 Electro‐Thermal Response Characterization

Connect both ends of the HHL to a DC‐regulated power supply (X08P3005, Toplia), energize the HHL under set voltage conditions, and use a thermal imager (ETS320, FLIR) to measure the temperature and capture thermal images.

#### 4.3.3 Electro‐Mechanical Response

The port of the HHL was connected to a DC‐regulated power supply (as above), and the sample was pre‐stretched in the uniaxial direction and placed on a straightedge for length calibration. An electrical pulse of 0.7 V was then applied, and the contraction was recorded with a camera.

#### 4.3.4 Multi‐Axial Stretching and Mechanical Output

The HHL was placed on a customized multi‐axial stretchable platform, stretched in the given axial direction, and loaded with a DC power supply for actuation, and its stress output was recorded in real time.

### 4.4 Robustness Testing

Mechanical testing of MSWZ was conducted under both dry conditions and in an artificial sweat environment. Prior to the test, the MSWZ was immersed in artificial sweat for 48 h. For uniaxial tensile testing, the strain was set to 5%–15% with 1,000 cycles. The MSWZ was subjected to both tests in both dry and sweat‐soaked conditions, and its mechanical robustness was evaluated through stress–strain curve analysis and shape recovery assessment.

### 4.5 FEA

The mechanical response of HHL under different tensile conditions was numerically simulated using FEA. The geometric model is based on experimental samples. The material properties are defined based on the hyperelastic intrinsic model, and the parameters are obtained from literature and experimental calibration. The model is divided into appropriate 3D solid units, and tensile displacements in different directions are imposed in the boundary conditions to simulate different tensile states. Nonlinear static analysis is used to solve the load‐displacement response and stress distribution, and the results are used to evaluate the deformable performance and adaptability of HHL under different loading modes.

### 4.6 Laboratory Animals and Ethical Approval

All ethical norms related to animal experimentation were strictly followed in this study. All experimental procedures involving animals were reviewed and approved by the institutional animal care and use committee of the University of Electronic Science and Technology of China (UESTC) (No. 106142025091534170) and the ethics committee of the Army Medical University (No. AMUWEC20265509), and were in accordance with the guidelines of the Animal Experimentation Center of the UESTC. The SD rats used in the experiments were purchased from Chengdu Dashuo Laboratory Animal Co. in China.

### 4.7 Wound Modeling Experiment

In this study, 6–8 weeks old male SD rats weighing 200–250 g were used, which were fasted for 12 h before the experiment and given free access to water. The rats were anesthetized with 2% isoflurane (Reward) inhalation anesthesia. After the anesthesia was stabilized, the backs of the rats were shaved and disinfected with iodophor and 75% ethanol. Circular wounds (approximately 10 mm in diameter) and linear wounds (10 mm in length) were made symmetrically on both sides of the spine using a scalpel to create wounds deep to the superficial fascial layer, respectively. As described above, spindle‐shaped wounds (dimensions: 15 mm × 9 mm) and oval‐shaped wounds (dimensions: 15 mm × 12 mm) were created on the backs of rats. After wound formation, adequate hemostasis was performed using an electrocoagulation hemostat, and MSWZ was subsequently applied according to the experimental grouping.

### 4.8 Histopathological Staining

Wound tissues were fixed in paraformaldehyde immediately after sampling, dehydrated by gradient ethanol, and embedded in paraffin. And sections were dewaxed and hydrated for HE, Masson, IHC, and IF staining, respectively.

#### 4.8.1 HE Staining

Sections were nucleated by hematoxylin staining, rinsed and differentiated by hydrochloric acid alcohol, returned to blue and stained with eosin. After dehydration, transparency, and sealing, tissue morphology and inflammatory cell infiltration were observed;

#### 4.8.2 Masson Staining

Sections were stained with iron hematoxylin for nuclei, and then sequentially stained with magenta complex, phosphomolybdic acid for differentiation, and bright green staining for assessing collagen fiber deposition and tissue remodeling.

#### 4.8.3 IHC

Sections were antigenically repaired, H_2_O_2_‐contained for endogenous peroxidase, and BSA‐containing. Subsequently, the primary antibody was added and incubated overnight, and the following day, the secondary antibody was added and incubated at room temperature. After DAB color development, hematoxylin re‐staining, and sealing of the sections for microscopic observation.

#### 4.8.4 IF

After deparaffinization and repair, the sections were closed with BSA, then incubated with primary antibody, added fluorescent‐labeled secondary antibody, and incubated at room temperature. The nuclei were restained by DAPI and sealed. A fluorescence microscope or a confocal microscope was used to observe and capture images. All sections were scanned in panoramic view by a digital section scanner (OLYMPUS, VS200+, JAPAN), and the results were analyzed using Image J (National Institutes of Health, USA).

### 4.9 Cell Compatibility Assay

L929 cells in the logarithmic growth phase were taken for the experiment. Sterile MSWZ was added into the culture medium, extracted for 24 h, and the supernatant was removed by a 0.22 µm filter membrane and prepared for use. Cells were cultured using the leachate in the experimental group and fresh medium in the control group. After treatment, the cells were fixed with 4% paraformaldehyde, permeabilized with 0.1% Triton X‐100, and blocked with 5% BSA. The cells were incubated with Vimentin primary antibody overnight and fluorescent secondary antibody the next day at room temperature and protected from light. After DAPI staining of the nuclei, the slices were washed with PBS and sealed. The relevant antibodies used in this study are shown in Table S1.

### 4.10 Statistical Analysis

Statistical analyses were performed using GraphPad Prism (version 9.5.0, GraphPad Software). Comparisons between two groups were conducted using two‐tailed unpaired t‐tests. For datasets involving more than two groups, one‐way ANOVA followed by Tukey's post hoc test was applied, while subgroup analyses were evaluated using two‐way ANOVA with Tukey's multiple comparison procedure. Graphical presentations were generated with Origin 2021 (OriginLab Corporation) and GraphPad Prism. Data are expressed as mean ± standard deviation, and differences were considered statistically significant at p < 0.05.

## Author Contributions

S. C. and G.Y. conceived the concept. G.Y., Y.L. and Y.Z. designed and supervised the research. Z. C., T. P., M. G., and D. F. provided laboratory assistance. S.C., G.Y., Z. C., S. Z., Z.Z., L. J., X.G., and C.Z. performed the experiments and data processing. P. L. and H. Y. participated in FEA. L. L. Participated in anesthesia and intraoperative monitoring of experimental animals. C. S. and G.Y. analyzed the data and wrote the manuscript. All authors reviewed and provided constructive feedback on the manuscript. S. C., G.Y., Z. C., and S. Z. equally contributed to this work. Correspondence and requests for materials should be addressed to Y. Z., G. Y., or Y. L.

## Conflicts of Interest

The authors declare no conflicts of interest.

## Supporting information




**Supporting File 1**: advs75744‐sup‐0001‐SuppMat.docx.

**Supporting File 2**: advs75744‐sup‐0002‐MovieS1.mp4.

**Supporting File 3**: advs75744‐sup‐0003‐MovieS2.mp4.

**Supporting File 4**: advs75744‐sup‐0004‐MovieS3.mp4.

**Supporting File 5**: advs75744‐sup‐0005‐MovieS4.mp4.

## Data Availability

The data that support the findings of this study are available from the corresponding author upon reasonable request.

## References

[1] A. J. Singer and R. A. Clark , “Cutaneous Wound Healing,” New England Journal of Medicine 341 (1999): 738–746, 10.1056/NEJM199909023411006. 10471461

[2] N. Charkoudian , “Skin Blood Flow in Adult Human Thermoregulation: How It Works, When It Does Not, and Why,” Mayo Clinic Proceedings 78 (2003): 603–612, 10.4065/78.5.603. 12744548

[3] O. A. Pena and P. Martin , “Cellular and Molecular Mechanisms of Skin Wound Healing,” Nature Reviews Molecular Cell Biology 25 (2024): 599–616, 10.1038/s41580-024-00715-1. 38528155

[4] X. Liu , Y. Sun , J. Wang , et al., “A Tough, Antibacterial and Antioxidant Hydrogel Dressing Accelerates Wound Healing and Suppresses Hypertrophic Scar Formation in Infected Wounds,” Bioactive Materials 34 (2024): 269–281. 38261887 10.1016/j.bioactmat.2023.12.019 PMC10794931

[5] N. Graves , C. J. Phillips , and K. Harding , “A Narrative Review of the Epidemiology and Economics of Chronic Wounds,” British Journal of Dermatology 187 (2022): 141–148, 10.1111/bjd.20692. 34549421

[6] M. Cui , Y. Jia , Z. Chen , et al., “Primary Closure and Prophylactic Antibiotics for Treatment of Traumatic Wounds Caused by Mammals, a Systematic Review and Meta‐analysis,” World Journal of Emergency Surgery 20 (2025): 48, 10.1186/s13017-025-00619-1. 40462162 PMC12135349

[7] W. Liu , L. Zu , S. Wang , et al., “Tailored Biomedical Materials for Wound Healing,” Burns & Trauma 11 (2023): tkad040, 10.1093/burnst/tkad040. 37899884 PMC10605015

[8] Y. Y. Li , S. F. Ji , X. B. Fu , Y. F. Jiang , and X. Y. Sun , “Biomaterial‐based Mechanical Regulation Facilitates Scarless Wound Healing with Functional Skin Appendage Regeneration,” Military Medical Research 11 (2024): 13. 38369464 10.1186/s40779-024-00519-6 PMC10874556

[9] H. Chen , R. Zhang , G. Zhang , et al., “Naturally Inspired Tree‐ring Structured Dressing Provides Sustained Wound Tightening and Accelerates Closure,” Advanced Materials 37 (2025): 2410845. 10.1002/adma.202410845 39533478

[10] N. Wang , J. D. Tytell , and D. E. Ingber , “Mechanotransduction at a Distance: Mechanically Coupling the Extracellular Matrix with the Nucleus,” Nature Reviews Molecular Cell Biology 10 (2009): 75–82, 10.1038/nrm2594. 19197334

[11] G. Yao , X. Mo , C. Yin , et al., “A Programmable and Skin Temperature–Activated Electromechanical Synergistic Dressing for Effective Wound Healing,” Science Advances 8 (2022): abl8379, 10.1126/sciadv.abl8379. PMC8791608 35080981

[12] M. Hosseini , J. Brown , K. Khosrotehrani , A. Bayat , and A. Shafiee , “Skin Biomechanics: A Potential Therapeutic Intervention Target to Reduce Scarring,” Burns & Trauma 10 (2022): tkac036, 10.1093/burnst/tkac036. 36017082 PMC9398863

[13] T. W. Thomsen , D. A. Barclay , and G. S. Setnik , “Basic Laceration Repair,” New England Journal of Medicine 355 (2006): 18, 10.1056/NEJMvcm064238. 17065633

[14] T. Tsujinaka , K. Yamamoto , J. Fujita , et al., “Subcuticular Sutures versus Staples for Skin Closure After Open Gastrointestinal Surgery: A Phase 3, Multicentre, Open‐label, Randomised Controlled Trial,” The Lancet 382 (2013): 1105–1112, 10.1016/S0140-6736(13)61780-8. 24075050

[15] J. H. Ko , I. H. Yang , M. S. Ko , E. Kamolhuja , and K. K. Park , “Do Zip‐Type Skin‐Closing Devices Show Better Wound Status Compared to Conventional Staple Devices In Total Knee Arthroplasty?,” International Wound Journal 14 (2017): 250–254, 10.1111/iwj.12596. 27019972 PMC7949581

[16] D. J. Leaper , “Traumatic and Surgical Wounds,” Bmj 332 (2006): 532–535, 10.1136/bmj.332.7540.532. 16513711 PMC1388134

[17] J. Yi , X. Ren , Y. Li , et al., “Rapid‐Response Water‐Shrink Films with High Output Work Density Based on Polyethylene Oxide and α‐Cyclodextrin for Autonomous Wound Closure,” Advanced Materials 36 (2024): 2403551, 10.1002/adma.202403551. 38837826

[18] L. Chang , H. Du , F. Xu , C. Xu , and H. Liu , “Hydrogel‐enabled Mechanically Active Wound Dressings,” Trends in Biotechnology 42 (2024): 31–42, 10.1016/j.tibtech.2023.06.004. 37453911

[19] J. Y. Hu , T. Wei , H. Zhao , et al., “Mechanically Active Adhesive and Immune Regulative Dressings for Wound Closure,” Matter 4 (2021): 2985–3000.

[20] Y. P. Dong , X. H. Zhang , Y. X. Chen , et al., “Stiff‐elastic" Binary Synergistic Fibrous Tape with Thermal‐Triggered Shrinkable and Shape Recoverable Performances for Wound Closure,” Advanced Functional Materials 34 (2024): 2402252.

[21] G. Theocharidis , H. Yuk , H. Roh , et al., “A Strain‐programmed Patch for the Healing of Diabetic Wounds,” Nature Biomedical Engineering 6 (2022): 1118–1133, 10.1038/s41551-022-00905-2. 35788686

[22] J. Wu , S. Yao , H. Zhang , et al., “Liquid Crystal Elastomer Metamaterials with Giant Biaxial Thermal Shrinkage for Enhancing Skin Regeneration,” Advanced Materials 33 (2021): 2106175. 10.1002/adma.202106175 34561930

[23] I. Atmaca and A. Yigit , “Predicting the Effect of Relative Humidity on Skin Temperature and Skin Wettedness,” Journal of Thermal Biology 31 (2006): 442–452, 10.1016/j.jtherbio.2006.03.003.

[24] N. C. Nowak , D. M. Menichella , R. Miller , and A. S. Paller , “Cutaneous Innervation in Impaired Diabetic Wound Healing,” Translational Research 236 (2021): 87–108, 10.1016/j.trsl.2021.05.003. 34029747 PMC8380642

[25] O. Barzilay and A. Gefen , “Be There or be Square: Should We Adopt Non‐Rectangular Dressing Shapes in Single‐Use Negative Pressure Wound Therapy?,” Journal of Tissue Viability 35 (2026): 100977, 10.1016/j.jtv.2025.100977. 41351949

[26] A. Lustig and A. Gefen , “Three‐Dimensional Shape‐Conformation Performances of Wound Dressings Tested in a Robotic Sacral Pressure Ulcer Phantom,” International Wound Journal 18 (2021): 670–680, 10.1111/iwj.13569. 33605541 PMC8450790

[27] Z. Chuanzhen , P. Jaeho , E. R. Samuel , et al., “Skin‐inspired Soft Bioelectronic Materials, Devices and Systems,” Nature Reviews Bioengineering 2 (2024): 671–690.

[28] D.‐H. Kim , N. Lu , R. Ma , et al., “Epidermal Electronics,” Science 333 (2011): 838–843, 10.1126/science.1206157. 21836009

[29] C. Wang , E. S. Sani , and W. Gao , “Wearable Bioelectronics for Chronic Wound Management,” Advanced Functional Materials 32 (2022): 2111022. 36186921 10.1002/adfm.202111022 PMC9518812

[30] J. Xin , L. Gao , W. Zhang , et al., “A Thermogalvanic Cell Dressing for Smart Wound Monitoring and Accelerated Healing,” Nature Biomedical Engineering 10 (2026): 80–93, 10.1038/s41551-025-01440-6. 40659833

[31] Y. Jiang , A. A. Trotsyuk , S. Niu , et al., “Wireless, Closed‐loop, Smart Bandage with Integrated Sensors and Stimulators for Advanced Wound Care and Accelerated Healing,” Nature Biotechnology 41 (2023): 652–662, 10.1038/s41587-022-01528-3. 36424488

[32] J. W. Song , H. Ryu , W. Bai , et al., “Bioresorbable, Wireless, and Battery‐free System for Electrotherapy and Impedance Sensing at Wound Sites,” Science Advances 9 (2023): ade4687, 10.1126/sciadv.ade4687. PMC9946359 36812305

[33] C. Xu and C. Wang , “A Stretchable Wireless Wearable Bioelectronic System for Multiplexed Monitoring and Combination Treatment of Infected Chronic Wounds,” Science Advances 9 (2023): adf7388. 10.1126/sciadv.adf7388 PMC10038347 36961905

[34] B. Kang , N. Zavanelli , G. N. Sue , et al., “A Flexible Skin‐Mounted Haptic Interface for Multimodal Cutaneous Feedback,” Nature Electronics 8 (2025): 818–830, 10.1038/s41928-025-01443-w.

[35] K.‐I. Jang , H. U. Chung , S. Xu , et al., “Soft Network Composite Materials with Deterministic and Bio‐Inspired Designs,” Nature Communications 6 (2015): 6566, 10.1038/ncomms7566. PMC4383007 25782446

[36] Q. Ma , H. Cheng , K.‐I. Jang , et al., “A Nonlinear Mechanics Model of Bio‐Inspired Hierarchical Lattice Materials Consisting of Horseshoe Microstructures,” Journal of the Mechanics and Physics of Solids 90 (2016): 179–202, 10.1016/j.jmps.2016.02.012. 27087704 PMC4831080

[37] J. Frenzel , E. P. George , A. Dlouhy , C. Somsen , M. F.‐X. Wagner , and G. Eggeler , “Influence of Ni on Martensitic Phase Transformations in NiTi Shape Memory Alloys,” Acta Materialia 58 (2010): 3444–3458, 10.1016/j.actamat.2010.02.019.

[38] Y. Ma , X. Feng , J. A. Rogers , Y. Huang , and Y. Zhang , “Design and Application of ‘J‐Shaped’ Stress–Strain Behavior in Stretchable Electronics: A Review,” Lab on a Chip 17 (2017): 1689–1704, 10.1039/C7LC00289K. 28470286 PMC5505255

[39] S. Medina‐Lombardero , C. Bain , L. Charlton , et al., “The Biomechanics of Wounds at Physiologically Relevant Levels: Understanding Skin's Stress‐shielding Effect for the Quantitative Assessment of Healing,” Mater Today Bio 25 (2024): 100963. 10.1016/j.mtbio.2024.100963 PMC10835282 38312802

[40] M. Pensalfini , E. Haertel , R. Hopf , M. Wietecha , S. Werner , and E. Mazza , “The Mechanical Fingerprint of Murine Excisional Wounds,” Acta Biomaterialia 65 (2018): 226–236, 10.1016/j.actbio.2017.10.021. 29031511

[41] N. Marsidi , S. A. M. Vermeulen , T. Horeman , and R. E. Genders , “Measuring Forces in Suture Techniques for Wound Closure,” Journal of Surgical Research 255 (2020): 135–143, 10.1016/j.jss.2020.05.033. 32543379

[42] M. G. Jeschke , F. M. Wood , E. Middelkoop , et al., “Scars,” Nature Reviews Disease Primers 9 (2023): 64, 10.1038/s41572-023-00474-x. 37973792

[43] C. Wang , E. Shirzaei Sani , C.‐D. Shih , et al., “Wound Management Materials and Technologies from Bench to Bedside and Beyond,” Nature Reviews Materials 9 (2024): 550–566, 10.1038/s41578-024-00693-y. PMC12176411 40535534

[44] D. S. Chen , B. Wang , T. H. Deng , et al., “Wearable Sensors Using Ionic Conductive Gels for Sweat Analysis and Sports Monitoring System Development,” Chemical Engineering Journal 530 (2026): 173024.

[45] R. C. Webb , A. P. Bonifas , A. Behnaz , et al., “Ultrathin Conformal Devices for Precise and Continuous Thermal Characterization of Human Skin,” Nature Materials 12 (2013): 938–944, 10.1038/nmat3755. 24037122 PMC3825211

[46] T. Q. Trung and N. E. Lee , “Flexible and Stretchable Physical Sensor Integrated Platforms for Wearable Human‐Activity Monitoringand Personal Healthcare,” Advanced Materials 28 (2016): 4338–4372, 10.1002/adma.201504244. 26840387

[47] R. M. Sarate , J. Hochstetter , M. Valet , et al., “Dynamic Regulation of Tissue Fluidity Controls Skin Repair During Wound Healing,” Cell 187 (2024): 5298–5315.e19, 10.1016/j.cell.2024.07.031. 39168124

[48] C. Rosendahl , M. Hishon , and B. N. Akay , “Shave versus Elliptical Biopsy for Melanoma Substantially Increases Re‐excision Area and Length,” Dermatologic Surgery 44 (2018): 731–733, 10.1097/DSS.0000000000001292. 28902029

[49] M. Nantel‐Battista and C. Murray , “Dermatologic Surgical Pearls: Enhancing the Efficacy of the Traditional Elliptical Excision,” Journal of Cutaneous Medicine and Surgery 19 (2015): 287–290, 10.2310/7750.2014.14095. 25775643

[50] A. Kimyai‐Asadi , L. H. Goldberg , A. Nemeth , et al., “Mohs Micrographic Surgery for Elliptical Excision of Skin Tumors: A Surgical and Histologic Study,” Dermatologic Surgery 30 (2004): 1317–1318. 10.1111/j.1524-4725.2004.30401.x 15458528

[51] R. Zou , F. Lin , C. Hao , D. Zhou , J. Liang , and H. Wang , “Assessment of Mathematical Model for Elliptical Excision: Solving the Doubt About Vertex Angle and Predicting Postoperative Wound Length,” BMC Surgery 23 (2023): 328, 10.1186/s12893-023-02234-w. 37891559 PMC10612151

[52] S. Schreml , R. M. Szeimies , L. Prantl , S. Karrer , M. Landthaler , and P. Babilas , “Oxygen in Acute and Chronic Wound Healing,” British Journal of Dermatology 163 (2010): 257–268, 10.1111/j.1365-2133.2010.09804.x. 20394633

[53] G. L. Semenza , “Targeting Hif‐1 for Cancer Therapy,” Nature Reviews Cancer 3 (2003): 721–732, 10.1038/nrc1187. 13130303

[54] G. Li , C.‐N. Ko , D. Li , et al., “A Small Molecule HIF‐1α Stabilizer that Accelerates Diabetic Wound Healing,” Nature Communications 12 (2021): 3363, 10.1038/s41467-021-23448-7. PMC8184911 34099651

[55] N. Ullrich , A. Schröder , J. Jantsch , G. Spanier , P. Proff , and C. Kirschneck , “The Role of Mechanotransduction Versus Hypoxia During Simulated Orthodontic Compressive Strain—An In Vitro Study of Human Periodontal Ligament Fibroblasts,” International Journal of Oral Science 11 (2019): 33, 10.1038/s41368-019-0066-x. 31685804 PMC6828658

[56] O. Ritsvall and S. Albinsson , “Emerging Role of Yap/Taz in Vascular Mechanotransduction and Disease,” Microcirculation 31 (2024): 12838, 10.1111/micc.12838. 38011540

[57] S. Dupont , L. Morsut , M. Aragona , et al., “Role of yap/taz in Mechanotransduction,” Nature 474 (2011): 179–183, 10.1038/nature10137. 21654799

[58] F. Sabeh , X.‐Y. Li , A. W. Olson , et al., “Mmp14‐Dependent Remodeling of the Pericellular–Dermal Collagen Interface Governs Fibroblast Survival,” Journal of Cell Biology 223 (2024): 202312091, 10.1083/jcb.202312091. PMC11244150 38990714

[59] C. A. Worthen , Y. Cui , J. S. Orringer , T. M. Johnson , J. J. Voorhees , and G. J. Fisher , “Cd26 Identifies a Subpopulation of Fibroblasts That Produce the Majority of Collagen During Wound Healing in human Skin,” Journal of Investigative Dermatology 140 (2020): 2515–2524.e3, 10.1016/j.jid.2020.04.010. 32407715 PMC7655599

[60] J. RUZICKA , M. GRAJCIAROVÁ , L. VISTEJNOVÁ , et al., “Hyperbaric Oxygen Enhances Collagen Iii Formation in Wound of Zdf Rat,” Physiological Research 70 (2021): 787–798, 10.33549/physiolres.934684. 34505531 PMC8820531

[61] F. S. Younesi , A. E. Miller , T. H. Barker , F. M. V. Rossi , and B. Hinz , “Fibroblast and Myofibroblast Activation in Normal Tissue Repair and Fibrosis,” Nature Reviews Molecular Cell Biology 25 (2024): 617–638, 10.1038/s41580-024-00716-0. 38589640

[62] L. Heskin , K. Bourke , and J. Kelly , “A Technique to Measure the Tension Across a Wound in Real Time During Wound Closure,” Journal of Plastic, Reconstructive & Aesthetic Surgery 71 (2018): 1216–1230, 10.1016/j.bjps.2018.05.028. 29936000

[63] R. Luo , J. Dai , J. Zhang , and Z. Li , “Accelerated Skin Wound Healing by Electrical Stimulation,” Advanced Healthcare Materials 10 (2021): 2100557, 10.1002/adhm.202100557. 33945225

[64] R. Yan , X. Zhang , H. Wang , et al., “Autonomous, Moisture‐Driven Flexible Electrogenerative Dressing for Enhanced Wound Healing,” Advanced Materials 37 (2025): 2418074, 10.1002/adma.202418074. 39962841

