# Supplementary material for: Multi‐Axis Stretchable Zippers for Personalized Wound Healing
Source: Adv Sci (Weinh). 2026 Jun 11;13(45):e75744. doi: 10.1002/advs.75744 (PMC13335945; doi:10.1002/advs.75744)
Supplement: Supplementary file 1 [file ADVS-13-e75744-s005.docx]

Multi-axis stretchable zippers for personalized wound healing

Siyuan Cai^1,2,3,4, †^, Guang Yao^2,3,6,7,^ *^, †^, Zijian Chen^1, †^, Shiqi Zhou^1, †^, Peisi Li^2^, Liheng Lin^5^, Huake Yang^1^, Ziyi Zhou^1^, Linbo, Jin^1^, Xingyi Gan^2^, Chenzheng Zhou^2^, Zhen Cai^4^, Taisong Pan^2^, Min Gao^2^, Dongli Fan^1^, Yuan Lin^2,3,6,7,^ *,Yiming Zhang^1,^ *

^1^Department of Plastic and Cosmetic Surgery, Xinqiao Hospital, Army Medical University, Chongqing 400037, China.

^2^School of Materials and Energy, University of Electronic Science and Technology of China, Chengdu 610054, Sichuan, China.

^3^State Key Laboratory of Electronic Thin Films and Integrated Devices, University of Electronic Science and Technology of China, Chengdu 610054, Sichuan, China.

^4^Department of Plastic Surgery, Sichuan Provincial People's Hospital, University of Electronic Science and Technology of China, Chengdu 610072, Sichuan, China.

^5^Department of Anesthesiology, The First People's Hospital of Longquanyi District, Chengdu 610199, Sichuan, China

^6^Shenzhen Institute for Advanced Study, University of Electronic Science and Technology of China, Shenzhen 518110, Guangzhou, China.

^7^Medico-Engineering Cooperation on Applied Medicine Research Center, University of Electronic Science and Technology of China, Chengdu 610054, Sichuan, China.

*Correspondence should be addressed to Y.Z. (zhangyiming@tmmu.edu.cn), G.Y. (gyao@uestc.edu.cn) or Y.L. (linyuan@uestc.edu.cn)

^†^ These authors contributed equally to this work.

The PDF file contains:

Figures S1-16

Table S1-S2

Movies S1-4

Legends for movies:

Movie S1. Rapid Electro-Mechanical Response Performance of HHL.

Movie S2. HHL's excellent mechanical shrinkage performance.

Movie S3. MSWZ mechanically closes wounds according to different wound morphologies.

Movie S4. Comparison of wound closure between mswz and surgical suturing.

**Figure S1.** Schematic diagram illustrating the principle of electrothermal contraction in the MSWZ. a) At the six ports of the MSWZ, vertical through-holes are present in the Ecoflex insulating film, filled with conductive silver paste. The Ni-Ti SMA and the PI/Cu conductive layer establish electrical connections at these ports (the six ports are connected to a DC power supply, divided into three positive and three negative terminals). b) Upon energization, the six ports form an electrical circuit within the Ni-Ti SMA. As current flows through the Ni-Ti SMA, it rapidly heats the material, triggering a phase transition and resulting in contraction.

**Figure S2.** Images of the uniaxial stretching process of HHL at different *θ*_HHL_

**Figure S3.** Uniaxial stretching FEA results of HHL at different *θ*_HHL_. a) *θ*_HHL_= 120^°^, b) *θ*_HHL_= 150^°^, c) *θ*_HHL_= 180^°^, and d) *θ*_HHL_= 210^°^.

**Figure S4.** HHL pre-stretched to the maximum level according to different wound morphology conditions.

**Figure S5.** Residual elongation of HHL according to different wound morphology conditions. a) linear wound, b) triangular wound, c) rectangular wound, and d) circular wound.

**Figure S6.** FEA results of out-of-plane buckling of HHL by maximal pre-stretching. a) Linear wound condition. b) Triangular wound condition. c) Rectangular wound condition. d) Circular wound condition.

**Figure S7.** Electro-thermal response of HHL under IR thermography.

**Figure S8**. Cyclic tensile fatigue testing of MSWZ. a) Schematic diagram of uniaxial cyclic tensile testing of MSWZ. b) Results of cyclic tensile testing of MSWZ in dry state and after immersion in artificial sweat (tensile strain: 5–15%, number of cycles: 1,000). (c-d) MSWZ maintains effective tensile and contraction performance even after cyclic testing: (c) dry state, (d) wet state.

**Figure S9.** Validation of MSWZ treatment for spindle-shaped wounds. a) Schematic illustration of MSWZ contracting to close a spindle-shaped wound after pre-stretching. b) Representative healing images of spindle-shaped wounds. c) Statistical histogram of wound closure rates. Data are presented as mean ± SD. Two-way ANOVA with Tukey's post hoc test was performed. N = 6 per group. P < 0.05 is considered statistically significant.

**Figure S10.** Validation of MSWZ treatment for oval-shaped wounds. a) Schematic illustration of MSWZ contracting to close a oval-shaped wound after pre-stretching. b) Representative healing images of oval-shaped wounds. c) Statistical histogram of wound closure rates. Data are presented as mean ± SD. Two-way ANOVA with Tukey's post hoc test was performed. N = 6 per group. P < 0.05 is considered statistically significant.

**Figure S11.** Revascularization within the wound in each group. a) IHC images of CD31 in wounds. b) Quantitative analysis of CD31, HPF: High Power Field. Data are presented as mean ± SD. One-way ANOVA with Tukey’s post hoc test was performed. Sample size N = 5; P < 0.05 was considered statistically significant.

**Figure S12.** Expression of YAP-associated mechanosensitive signaling in wound tissue. a) Representative IF of wound tissue: YAP is shown in green, DAPI-stained cell nuclei are shown in blue, and vimentin is shown in red. b) Quantitative analysis of YAP expression within DAPI-defined nuclear regions. c) Quantitative analysis of YAP expression within vimentin-positive regions. Data are presented as mean ± SD. One-way ANOVA with Tukey’s post hoc test was performed. Sample size N = 5; P < 0.05 was considered statistically significant.

**Figure S13.** Col I/Col III ratios within wounds in each group. Data are presented as mean ± SD. One-way ANOVA with Tukey’s post hoc test was performed. Sample size N = 5; P < 0.05 was considered statistically significant.

**Figure S14.** Expression of activated myofibroblasts in wounds. a) IHC images of α-SMA in wounds. b) Quantitative analysis of α-SMA. Data are presented as mean ± SD. One-way ANOVA with Tukey’s post hoc test was performed. Sample size N = 5; P < 0.05 was considered statistically significant.

**Figure S15.** A radar chart comparing the performance of MSWZ with that of other studies, with the scores for each performance metric taken from Table S1.

**Figure S16**. Schematic diagram of the multifunctional extension design of the MSWZ. a) Schematic diagram of the MSWZ integrating electrical stimulation and an antimicrobial coating for the treatment of chronic wounds (such as diabetic or infected wounds). b) The multi-functional MSWZ features stretchable Mo electrodes and an antimicrobial hydrogel coating.

**References**

Ref. 1. Chen H, Zhang R, Zhang G, et al. Naturally Inspired Tree-Ring Structured Dressing Provides Sustained Wound Tightening and Accelerates Closure. Adv Mater. 2025;37(3):e2410845. doi:10.1002/adma.202410845

Ref. 2. Theocharidis G, Yuk H, Roh H, et al. A strain-programmed patch for the healing of diabetic wounds. Nat Biomed Eng. 2022;6(10):1118-1133. doi:10.1038/s41551-022-00905-2

Ref. 3. Wu J, Yao S, Zhang H, et al. Liquid Crystal Elastomer Metamaterials with Giant Biaxial Thermal Shrinkage for Enhancing Skin Regeneration. Adv Mater. 2021;33(45):e2106175. doi:10.1002/adma.202106175

Ref. 4. Nowak NC, Menichella DM, Miller R, et al. Cutaneous innervation in impaired diabetic wound healing. Transl Res. 2021;236:87-108. doi:10.1016/j.trsl.2021.05.003

Ref. 5. İbrahim Atmaca, Abdulvahap Yiğit. Predicting the effect of relative humidity on skin temperature and skin wettedness. JOURNAL OF THERMAL BIOLOGY. 2006;31(5):442-452. doi:10.1016/j.jtherbio.2006.03.003

Ref. 6. Nowak NC, Menichella DM, Miller R, et al. Cutaneous innervation in impaired diabetic wound healing. Transl Res. 2021;236:87-108. doi:10.1016/j.trsl.2021.05.003
